# A scheme for realizing nonreciprocal interlayer coupling in bilayer topological systems

**DOI:** 10.1007/s12200-023-00094-z

**Published:** 2023-11-27

**Authors:** Xiaoxiao Wang, Ruizhe Gu, Yandong Li, Huixin Qi, Xiaoyong Hu, Xingyuan Wang, Qihuang Gong

**Affiliations:** 1 State Key Laboratory for Mesoscopic Physics and Department of Physics, Collaborative Innovation Center of Quantum Matter & Frontiers Science Center for Nano-Optoelectronics, Beijing Academy of Quantum Information Sciences, Peking University, Beijing, 100871 China; 2 Peking University Yangtze Delta Institute of Optoelectronics, Nantong, 226010 China; 3 Collaborative Innovation Center of Extreme Optics, Shanxi University, Taiyuan, 030006 China; 4 Hefei National Laboratory, Hefei, 230088 China; 5 College of Mathematics and Physics, Beijing University of Chemical Technology, Beijing, 100029 China

**Keywords:** Nonreciprocal, Bilayer, Interlayer coupling, Topological photonics

## Abstract

Nonreciprocal interlayer coupling is difficult to practically implement in bilayer non-Hermitian topological photonic systems. In this work, we identify a similarity transformation between the Hamiltonians of systems with nonreciprocal interlayer coupling and on-site gain/loss. The similarity transformation is widely applicable, and we show its application in one- and two-dimensional bilayer topological systems as examples. The bilayer non-Hermitian system with nonreciprocal interlayer coupling, whose topological number can be defined using the gauge-smoothed Wilson loop, is topologically equivalent to the bilayer system with on-site gain/loss. We also show that the topological number of bilayer non-Hermitian C_6v_-typed domain-induced topological interface states can be defined in the same way as in the case of the bilayer non-Hermitian Su–Schrieffer–Heeger model. Our results show the relations between two microscopic provenances of the non-Hermiticity and provide a universal and convenient scheme for constructing and studying nonreciprocal interlayer coupling in bilayer non-Hermitian topological systems. This scheme is useful for observation of non-Hermitian skin effect in three-dimensional systems.

**Graphical Abstract:**

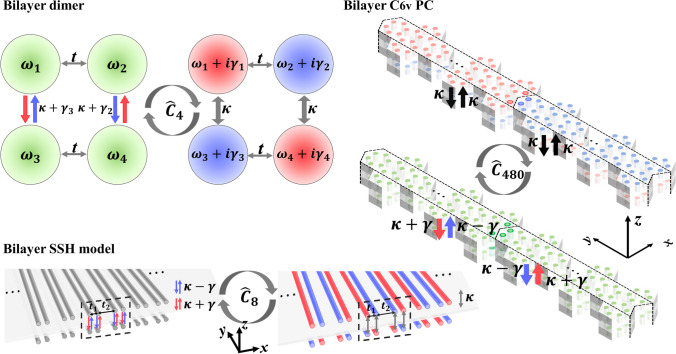

**Supplementary Information:**

The online version contains supplementary material available at 10.1007/s12200-023-00094-z.

## Introduction

Non-Hermitian systems are constructed by introducing gain-and-loss distributions [1, 2] or nonreciprocal interactions [3, 4], illustrating a good deal of unusual physics [5]. Nonreciprocal (anisotropic) coupling, is characterized by unbalanced couplings ${\kappa }_{ab}\ne {\kappa }_{ba}$ between two lattice sites *a* and *b* [4]. Where ${\kappa }_{ab}\ne {\kappa }_{ba}^{*}$ means that the mode amplitude undergoes gain or loss while couple between lattice sites *a* and *b* [6, 7]. Systems that break Lorentz reciprocity are nonreciprocal and prevent light from retracing the forward path [8, 9]. Nonreciprocity exists in topologically protected unidirectional edge states of topological photonics [10, 11]. Introducing nonreciprocal coupling into non-Hermitian topological photonics leads to intriguing phenomena [12], including non-Hermitian skin effect [3, 4, 13], higher-order exceptional points (EPs) [6], revised bulk-boundary correspondence [14], and new definitions of topological invariants [15].

Nonreciprocal systems were initially based on magneto-optical materials [16]. Recently, several approaches have been developed to generate nonreciprocity, including parity-time-symmetric nonlinear cavities [17], use of energy loss [18], spatial–temporal modulation [19, 20], and metamaterials [21]. However, these principles for implementing nonreciprocal interlayer coupling have practical difficulties, particularly in topological photonic systems because they may lose original topological properties after adding nonreciprocity [22].

In this paper, we provide a scheme for realizing the nonreciprocal interlayer coupling system by constructing on-site gain/loss in bilayer non-Hermitian topological systems. We reveal similarity transformations between nonreciprocal interlayer coupling and on-site gain/loss in the one-dimensional bilayer Su–Schrieffer–Heeger (SSH) model and two-dimensional bilayer C_6v_ topological photonic crystal (PC). The similarity transformations reveal that novel behaviors like delocalization [23], skin effect [4, 24], and breakdown of the conventional bulk-boundary correspondence [14] are generic non-Hermitian phenomena not tied to a specific microscopic provenance of the non-Hermiticity [5]. The topological number of the bilayer nonreciprocal interlayer coupling system, defined using a gauge-smoothed Wilson loop, is equal to that of the bilayer on-site gain-or-loss system. Topological phase transitions and parity-time-phase transitions of the non-Hermitian topological states occur by modulating the strength of nonreciprocal interlayer coupling or on-site gain/loss quantity. These results have great potential applications in reconfigurable laser arrays [23, 25–27], and for studying non-Hermitian topological physics, such as non-Hermitian band topology [14].

## Nonreciprocity-induced topological phase transition

**Fig. 1:**
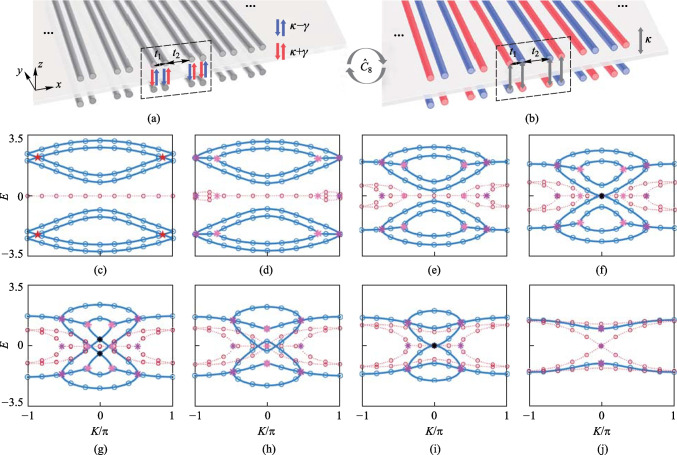
Schematic of bilayer non-Hermitian SSH model for **a** passive waveguide arrays with nonreciprocal interlayer coupling strength $\kappa +\gamma$ (red arrows) and $\kappa -\gamma$ (blue arrows), and **b** gain (red) and lossy (blue) waveguides. **c** − **j** Comparisons of the bulk bands of bilayer non-Hermitian SSH model using parameters ${t}_{1}=2, {t}_{2}=1, \kappa =0.2$, and $\gamma =0$ for **c**, $\gamma =0.5$ for **d**, $\gamma =1.25$ for **e**, $\gamma =1.497$ for **f**, $\gamma =1.697$ for **g**, $\gamma =1.8$ for **h**, $\gamma =1.96$ for **i**, and $\gamma =2.2$ for **j**. The blue solid (red dot) lines indicate the real (imaginary) part of eigenvalues of ${\widehat{H}}_{\mathrm{SSH}-\mathrm{PBC}}^{\mathrm{nonrecip}}(K)$, and the blue (red) discrete circles indicate the real (imaginary) part of eigenvalues of ${\widehat{H}}_{\mathrm{SSH}-\mathrm{PBC}}^{\mathrm{GL}}\left(K\right)$

The bilayer non-Hermitian SSH model [28] is constructed by stacked nonreciprocal interlayer coupling photonic waveguide arrays (Fig. 1a). The alternating distance between in-layer nearest-neighbor waveguide determines ${t}_{1}$ (short hopping) and ${t}_{2}$ (long hopping) [29–31]. Following coupled-mode theory under tight-binding approximation and applying Fourier transformation [6, 30], the Bloch Hamiltonian of the unit cell (black dotted box) under periodic boundary conditions (PBCs) is

1 $${\widehat{H}}_{\mathrm{SSH}-\mathrm{PBC}}^{\mathrm{nonrecip}}\left(K\right)=\left[\begin{array}{cc}{H}_{m}& {H}_{12}\\ {H}_{21}& {H}_{m}\end{array}\right],$$

where $K$ is Bloch wave vector. ${H}_{m}$ is the Hamiltonian of monolayer SSH model. ${H}_{12}$ and ${H}_{21}$ are nonreciprocal interlayer coupling matrices. See Appendix A for the forms of ${H}_{m}$, ${H}_{12}$, and ${H}_{21}$. We apply a similarity transformation to ${\widehat{H}}_{\mathrm{SSH}-\mathrm{PBC}}^{\mathrm{nonrecip}}\left(K\right):$

2 $${\widehat{\mathcal{C}}}_{8}{\widehat{H}}_{\mathrm{SSH}-\mathrm{PBC}}^{\mathrm{GL}}\left(K\right){\widehat{\mathcal{C}}}_{8}^{-1}={\widehat{H}}_{\mathrm{SSH}-\mathrm{PBC}}^{\mathrm{nonrecip}}\left(K\right),\space {\widehat{\mathcal{C}}}_{8}=\frac{1}{\sqrt{2}}\left({\widehat{\sigma }}_{x}-{\mathrm{i}\widehat{\sigma }}_{0}\right)\otimes {\widehat{I}}_{4},$$

where ${\widehat{\sigma }}_{0}$ and ${\widehat{\sigma }}_{x,y,z}$ are two-by-two identity matrix and Pauli matrix, and ${\widehat{I}}_{4}$ is a four-by-four identity matrix. The Bloch Hamiltonian of bilayer on-site gain-and-loss SSH model ${\widehat{H}}_{\mathrm{SSH}-\mathrm{PBC}}^{\mathrm{GL}}(K)$ is obtained as shown in Appendix A. *κ* is isotropic interlayer hopping (IIH) (gray arrows in Fig. 1b). The gain (and loss) strengths in gain (and lossy) waveguides are γ (Fig. 1b) (See Appendix B for generalized derivations with arbitrary gain/loss).When $K=0$, the eight periodic-boundary-condition eigenvalues are

3 $${E}_{\pm \pm \pm }^{\mathrm{SSH}}=\pm {t}_{2}\pm \sqrt{{({t}_{1}\pm \kappa )}^{2}-{\gamma }^{2}.}$$

Figures 1c–j compare the bulk bands given by ${\widehat{H}}_{\mathrm{SSH}-\mathrm{PBC}}^{\mathrm{nonrecip}}(K)$ and ${\widehat{H}}_{\mathrm{SSH}-\mathrm{PBC}}^{\mathrm{GL}}(K)$. When $\gamma =0$, there are four intersections (red pentagrams) (Fig. 1c). When $\gamma =0.5$, an intersection becomes two EPs (magenta and pink crosses), which move away from each other along the first Brillouin zone (FBZ) as *γ* increases, while the complex energy region expands from the intersection to both sides until the edge of the FBZ (Fig. 1d). Then all EPs begin to move toward $K=0$, while the complex energy region expands from edge to center of the FBZ (Fig. 1e). The real part of bands (RPBs) moves toward zero energy, and the central gap closes when $\upgamma =\sqrt{{({t}_{1}-\kappa )}^{2}-{{t}_{2}}^{2}}$ (Fig. 1f). The RPBs approach and form two central degenerate points (DPs) when $\upgamma =\sqrt{{t}_{1}^{2}+{\kappa }^{2}-{t}_{2}^{2}-{t}_{1}^{2}{\kappa }^{2}/{t}_{2}^{2}}$ (black crosses) (Fig. 1g). When $\upgamma ={t}_{1}-\kappa$, two EPs merge into one EP at $K=0$ (Fig. 1h). The RPBs form a central DP when $\upgamma =\sqrt{{({t}_{1}+\kappa )}^{2}-{{t}_{2}}^{2}}$ (Fig. 1i). When $\upgamma ={t}_{1}+\kappa$, the other two EPs merge into one EP at $K=0$ (Fig. 1j).

Hermitian SSH model is topologically nontrivial for ${t}_{1}<{t}_{2}$ and trivial for ${t}_{1}>{t}_{2}$. However, the bilayer structure can be changed from trivial phase to topologically nontrivial phase by increasing non-Hermitian quantities. The topological number of the bilayer non-Hermitian system is defined by the winding number [32, 33], which can be calculated using a gauge-smoothed Wilson loop [33, 34]:

4 $${Q}_{m}^{\mathrm{GL}}\approx \frac{1}{2\uppi }\mathrm{arg}[\langle {\chi }_{m}^{\mathrm{GL}}({K}_{0})|{\phi }_{m}^{\mathrm{GL}}\left({K}_{1})\right.\rangle \langle {\chi }_{m}^{\mathrm{GL}}\left({K}_{1}\right)|{\phi }_{m}^{\mathrm{GL}}\left({K}_{2}\right)\rangle \cdots \langle {\chi }_{m}^{\mathrm{GL}}\left({K}_{N-1}\right)|{\phi }_{m}^{\mathrm{GL}}\left({K}_{0}\right)\rangle ] \space (\mathrm{mod }1),$$

where $\left|{\phi }_{m}^{\mathrm{GL}}\left({K}_{i}\right)\right.\rangle$ and $\left|{\chi }_{m}^{\mathrm{GL}}\left({K}_{i}\right)\right.\rangle$ are the *m*th ($m=1,\dots ,8$) right and left eigenstates of ${\widehat{H}}_{\mathrm{SSH}-\mathrm{PBC}}^{\mathrm{GL}}\left({K}_{i}\right)$. ${K}_{i}$ ($i=0, 1, 2,\dots , N$) is discrete Bloch wave vector, and $-\uppi ={K}_{0}<{K}_{1}<{K}_{2}<\cdots <{K}_{N}=\uppi$, where *N* is a large integer number. Given the relation between the *m*th right and left eigenstates of ${\widehat{H}}_{\mathrm{SSH}-\mathrm{PBC}}^{\mathrm{GL}}\left({K}_{i}\right)$ and ${\widehat{H}}_{\mathrm{SSH}-\mathrm{PBC}}^{\mathrm{nonrecip}}\left({K}_{i}\right)$ (See Appendix C for the deduced process.):

5 $$\left|{\phi }_{m}^{\mathrm{GL}}\left({K}_{i+1}\right)\right.\rangle ={\widehat{\mathcal{C}}}_{8}^{-1}\left|{\phi }_{m}^{\mathrm{nonrecip}}\left({K}_{i+1}\right)\right.\rangle ,$$

6 $$\left|{\chi }_{m}^{\mathrm{GL}}\left({K}_{i}\right)\right.\rangle ={\widehat{\mathcal{C}}}_{8}^{-1}\left|{\chi }_{m}^{\mathrm{nonrecip}}\left({K}_{i}\right)\right.\rangle .$$

The Hermitian conjugate form of Eq. (6) is

7 $$\left.\langle {\chi }_{m}^{\mathrm{GL}}\left({K}_{i}\right)\right|=\left.\langle {\chi }_{m}^{\mathrm{nonrecip}}\left({K}_{i}\right)\right|{\widehat{\mathcal{C}}}_{8}.$$

**Fig. 2:**
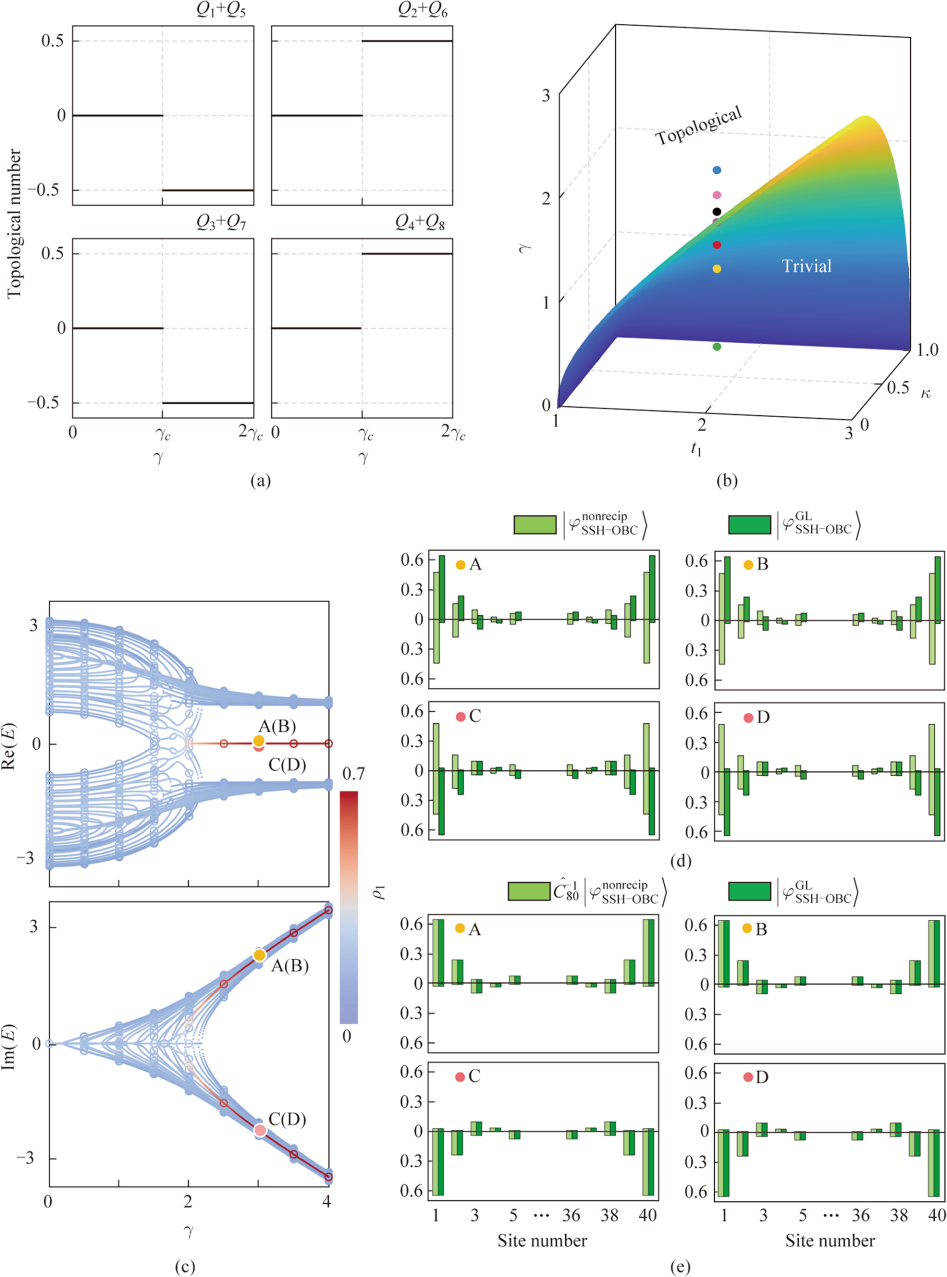
**a** Four topological numbers of the system: ${Q}_{1}+{Q}_{5}$, ${Q}_{2}+{Q}_{6}$, ${Q}_{3}+{Q}_{7}$, and ${Q}_{4}+{Q}_{8}$. **b** Topological phase diagram of non-Hermitian term γ, IIH term *κ*, and in-layer coupling ${t}_{1}$, which are normalized using ${t}_{2}$. Seven dots in various colors are highlighted, which correspond to the seven γ values in Fig. 1d–j. **c** Comparison of the eigenvalues of ${\widehat{H}}_{\mathrm{SSH}-\mathrm{OBC}}^{\mathrm{nonrecip}}$ (solid lines) and ${\widehat{H}}_{\mathrm{SSH}-\mathrm{OBC}}^{\mathrm{GL}}$ (discrete circles). **d** Comparison of $|{\varphi }_{\mathrm{SSH}-\mathrm{OBC}}^{\mathrm{nonrecip}}\rangle$ and $|{\varphi }_{\mathrm{SSH}-\mathrm{OBC}}^{\mathrm{GL}}\rangle$ for the four representative zero-energy edge states. **e** Comparison of ${\widehat{\mathcal{C}}}_{80}^{-1}|{\varphi }_{\mathrm{SSH}-\mathrm{OBC}}^{\mathrm{nonrecip}}\rangle$ and $|{\varphi }_{\mathrm{SSH}-\mathrm{OBC}}^{\mathrm{GL}}\rangle$ for the four representative states. Above (below) the horizontal line is normalized field distributions of the first (second) layer. Only five sites at the boundaries of each layer are shown

By multiplying Eq. (7) with Eq. (5), we get $\langle {\chi }_{m}^{\mathrm{GL}}\left({K}_{i}\right)|{\phi }_{m}^{\mathrm{GL}}\left({K}_{i+1}\right)\rangle =\left.\langle {\chi }_{m}^{\mathrm{nonrecip}}\left({K}_{i}\right)\right|{\phi }_{m}^{\mathrm{nonrecip}}({K}_{i+1})\rangle$, through which the gain-and-loss system is topologically equivalent to the nonreciprocal interlayer coupling system. ${\widehat{H}}_{\mathrm{SSH}-\mathrm{PBC}}^{\mathrm{nonrecip}}\left(K\right)$ has chiral symmetry ${\widehat{H}}_{\mathrm{SSH}-\mathrm{PBC}}^{\mathrm{nonrecip}}\left(K\right)=-\widehat{\varsigma }{{\widehat{H}}_{\mathrm{SSH}-\mathrm{PBC}}^{\mathrm{nonrecip}}\left(K\right)}^{*}{\widehat{\varsigma }}^{-1}$, where $\widehat{\varsigma }={\widehat{\sigma }}_{z}\otimes {\widehat{\sigma }}_{0}\otimes {\widehat{\sigma }}_{z}$. ${E}_{m}$ is the *m*th band, sorting the RPBs in ascending order. ${E}_{n}$ and ${E}_{m}$ satisfy chiral symmetry for ${E}_{n}=-{{E}_{m}}^{*}$. ${E}_{j}$ forms EPs with ${E}_{m}$. For each *m*, we add ${Q}_{n}^{B}$ and ${Q}_{j}^{B}$, and obtain four numbers characterizing topological number of the system (Fig. 2a). The four numbers change from 0 mod 1 to $\pm 0.5$ mod 1 at ${\gamma }_{c}=1.697$, indicating a topological phase transition. The bilayer non-Hermitian system is topologically nontrivial when $\gamma >{\gamma }_{c}$ and trivial when $\gamma <{\gamma }_{c}$, where ${\gamma }_{c}=\sqrt{{t}_{1}^{2}+{\kappa }^{2}-{t}_{2}^{2}-{t}_{1}^{2}{\kappa }^{2}/{t}_{2}^{2}}$ (${t}_{1}>{t}_{2}\ge \kappa$) is the topological phase boundary in the phase diagram (Fig. 2b).

Under open boundary conditions (OBCs), the waveguide array in each layer has 40 waveguides. Figure 2c compares real parts (RPs) and imaginary parts (IPs) of the open-boundary-condition *E* − *γ* relation given by ${\widehat{H}}_{\mathrm{SSH}-\mathrm{OBC}}^{\mathrm{nonrecip}}$ and ${\widehat{H}}_{\mathrm{SSH}-\mathrm{OBC}}^{\mathrm{GL}}$ using the same parameters as above. The energy bands in Fig. 2c are colored according to the ratio, ${\rho }_{1}$, of the sum of field intensity (SoFI) of the four sites at the boundaries of the bilayer chain to SoFI of all sites. ${\rho }_{1}$ describes the degree of locality of the edge states’ normalized field distributions. When $\gamma <1.697$, there are only bulk states. When $\gamma >1.697$, the RPs of the eigenvalues of the two pairs of degenerate topological edge states are close to zero, and the IPs of the eigenvalues are opposite. Figure 2d compares the normalized field distributions of four representative edge states of ${\widehat{H}}_{\mathrm{SSH}-\mathrm{OBC}}^{\mathrm{nonrecip}}$ and ${\widehat{H}}_{\mathrm{SSH}-\mathrm{OBC}}^{\mathrm{GL}}$ when $\gamma =3$. The edge states of ${\widehat{H}}_{\mathrm{SSH}-\mathrm{OBC}}^{\mathrm{GL}}$ are localized at boundaries of the SSH chain in the first (second) layer if the IPs of corresponding eigenvalues are positive (negative). The transformation matrix between ${\widehat{H}}_{\mathrm{SSH}-\mathrm{OBC}}^{\mathrm{nonrecip}}$ and ${\widehat{H}}_{\mathrm{SSH}-\mathrm{OBC}}^{\mathrm{GL}}$ is ${\widehat{\mathcal{C}}}_{80}=\frac{1}{\sqrt{2}}({\widehat{\sigma }}_{x}-{\mathrm{i}\widehat{\sigma }}_{0})\otimes {\widehat{I}}_{40}$. $|{\varphi }_{\mathrm{SSH}-\mathrm{OBC}}^{\mathrm{GL}}\rangle ={\widehat{\mathcal{C}}}_{80}^{-1}|{\varphi }_{\mathrm{SSH}-\mathrm{OBC}}^{\mathrm{nonrecip}}\rangle$ is shown in Fig. 2e, where $|{\varphi }_{\mathrm{SSH}-\mathrm{OBC}}^{\mathrm{nonrecip}}\rangle$ and $|{\varphi }_{\mathrm{SSH}-\mathrm{OBC}}^{\mathrm{GL}}\rangle$ are the eigenstates of ${\widehat{H}}_{\mathrm{SSH}-\mathrm{OBC}}^{\mathrm{nonrecip}}$ and ${\widehat{H}}_{\mathrm{SSH}-\mathrm{OBC}}^{\mathrm{GL}}$. The bilayer non-Hermitian SSH model defined by waveguide arrays can be fabricated inside glasses using femtosecond-laser direct writing techniques [35–37]. A re-exposure technique can be applied to introduce point scatterers inside waveguides, making the system be non-Hermitian [38].

## Nonreciprocity-induced topological interface states

**Fig. 3:**
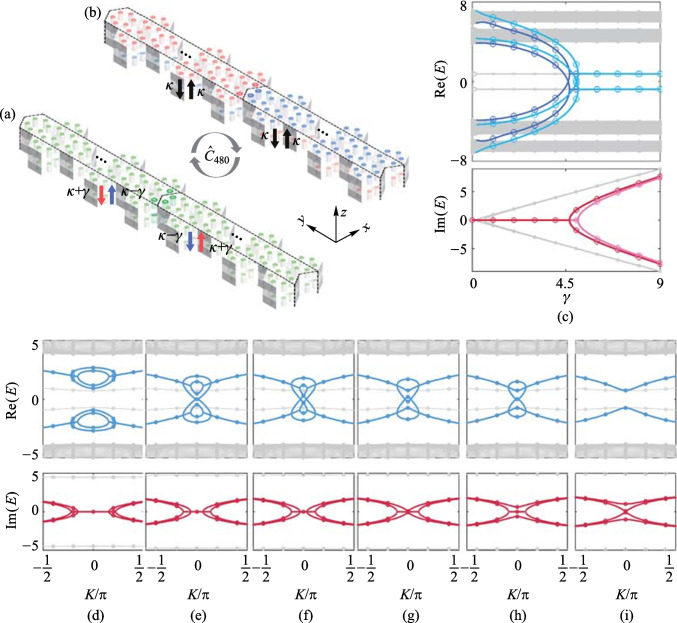
Bilayer supercell with non-Hermitian domain walls constructed by **a** nonreciprocal interlayer coupling strength $\kappa +\gamma$ (red arrows) and $\kappa -\gamma$ (blue arrows), and **b** on-site gain (red) and loss (blue). **c** Comparison of the eigenvalues of ${\widehat{H}}_{\mathrm{C}6-\mathrm{PBC}}^{\mathrm{nonrecip}}\left(K\right)$ (solid line) and ${\widehat{H}}_{\mathrm{C}6-\mathrm{PBC}}^{\mathrm{GL}}\left(K\right)$ (discrete circles). **d** − **i** Comparison of parts of the projected bands of ${\widehat{H}}_{\mathrm{C}6-\mathrm{PBC}}^{\mathrm{nonrecip}}\left(K\right)$ (solid line) and ${\widehat{H}}_{\mathrm{C}6-\mathrm{PBC}}^{\mathrm{GL}}\left(K\right)$ (discrete circles) with ${t}_{1}=5$, ${t}_{2}=1$, $\kappa =0.05$, and $\gamma =4.500$ for **d**, $\gamma =4.767$ for **e**, $\gamma =4.808$ for **f**, $\upgamma =4.831$ for **g**, $\gamma =4.870$ for **h**, and $\gamma =4.934$ for **i**

Given the C_6v_ PC with six sites per unit cell [39], a topologically trivial or nontrivial bandgap is opened when intercell (${t}_{1}$) and intracell (${t}_{2}$) nearest-neighbor couplings are not equal [39, 40]. With PBCs (OBCs) applied in the *x* (*y*) direction, the bilayer supercell of C_6v_ topologically nontrivial PC (${t}_{1}>{t}_{2}$) with zigzag-type domain walls [41–43] consists of 40 unit cells along *y* direction per layer. The non-Hermitian domain walls are constructed by nonreciprocal interlayer coupling (Fig. 3a) and on-site gain–loss (Fig. 3b).

Using tight-binding approximation and Bloch theorem of periodic lattice, the Hamiltonian of the bilayer nonreciprocal interlayer coupling supercell is [44]

8 $${\widehat{H}}_{\mathrm{C}6-\mathrm{PBC}}^{\mathrm{nonrecip}}\left(K\right)={\widehat{\sigma }}_{0}\otimes {H}_{\mathrm{mono}}+\kappa {\widehat{\sigma }}_{x}\otimes {\widehat{\sigma }}_{0}\otimes {\widehat{I}}_\frac{N}{2}+{\mathrm{i}\gamma \widehat{\sigma }}_{y}\otimes {\widehat{\sigma }}_{z}\otimes {\widehat{I}}_\frac{N}{2},$$

where ${H}_{\mathrm{mono}}$ is the Hamiltonian of monolayer supercell without gain or loss, and *κ* denotes IIH. After the similarity transformation is applied to ${\widehat{H}}_{\mathrm{C}6-\mathrm{PBC}}^{\mathrm{nonrecip}}\left(K\right)$ with ${\widehat{\mathcal{C}}}_{480}=\frac{1}{\sqrt{2}}({\widehat{\sigma }}_{x}-{\mathrm{i}\widehat{\sigma }}_{0})\otimes {\widehat{I}}_{240}$, the Hamiltonian of the bilayer on-site gain-and-loss supercell is

9 $${\widehat{H}}_{\mathrm{C}6-\mathrm{PBC}}^{\mathrm{GL}}\left(K\right)=\left[\begin{array}{cc}{H}_{\mathrm{GL}}& \kappa {\widehat{I}}_{N}\\ \kappa {\widehat{I}}_{N}& {H}_{\mathrm{LG}}\end{array}\right],$$

where ${H}_{\mathrm{GL}}$ and ${H}_{\mathrm{LG}}$ are the Hamiltonians of the first layer and second layer with non-Hermitian domain walls, respectively. ${\widehat{I}}_{N}$ (${\widehat{I}}_{N/2}$) is $N\times N$ ($\frac{N}{2}\times \frac{N}{2}$) identity matrix ($N=240$). See Appendix D for the forms of ${H}_{\mathrm{mono}}$, ${H}_{\mathrm{GL}}$, and ${H}_{\mathrm{LG}}$.

**Fig. 4:**
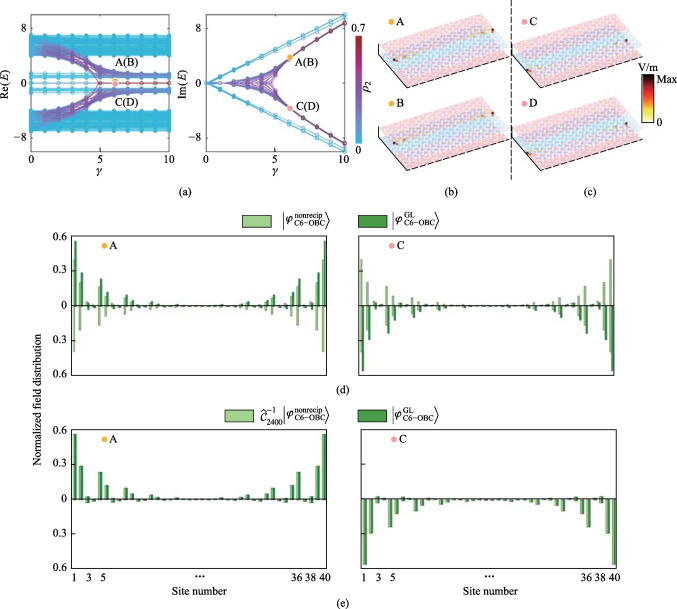
**a** Comparison of the eigenvalues of ${\widehat{H}}_{\mathrm{C}6-\mathrm{OBC}}^{\mathrm{nonrecip}}$ (solid line) and ${\widehat{H}}_{\mathrm{C}6-\mathrm{OBC}}^{\mathrm{GL}}$ (discrete circles). Normalized field distributions of four representative edge of interface states of ${\widehat{H}}_{\mathrm{C}6-\mathrm{OBC}}^{\mathrm{GL}}$ when $\gamma =6$, and the IPs of corresponding eigenvalues are positive **b** and negative **c**. **d** Comparison of $|{\varphi }_{\mathrm{C}6-\mathrm{OBC}}^{\mathrm{nonrecip}}\rangle$ and $|{\varphi }_{\mathrm{C}6-\mathrm{OBC}}^{\mathrm{GL}}\rangle$ for the two representative edge of interface states. **e** Comparison of ${\widehat{\mathcal{C}}}_{2400}^{-1}|{\varphi }_{\mathrm{C}6-\mathrm{OBC}}^{\mathrm{nonrecip}}\rangle$ and $|{\varphi }_{\mathrm{C}6-\mathrm{OBC}}^{\mathrm{GL}}\rangle$ for the two representative edge of interface states. Above (below) the horizontal line is normalized field distributions of the first (second) layer. Only 40 sites around the domain wall of both layers are shown.

Figure 3c compares the periodic-boundary-condition *E* − *γ* relation given by ${\widehat{H}}_{\mathrm{C}6-\mathrm{PBC}}^{\mathrm{nonrecip}}\left(K\right)$ and ${\widehat{H}}_{\mathrm{C}6-\mathrm{PBC}}^{\mathrm{GL}}\left(K\right)$ using parameters ${t}_{1}=5$, ${t}_{2}=1$, $\kappa =0.05$, and $K=0$. The eight eigenvalues whose RPs vary with *γ* are indicated in blue, and the corresponding IPs are indicated in red, as is the case for the bilayer non-Hermitian SSH model in Appendix A. Figures 3d − i compare parts of the projected bands given by ${\widehat{H}}_{\mathrm{C}6-\mathrm{PBC}}^{\mathrm{nonrecip}}\left(K\right)$ and ${\widehat{H}}_{\mathrm{C}6-\mathrm{PBC}}^{\mathrm{GL}}\left(K\right)$ with different *γ*. Eight bands, which are new topological interface states localized at the bilayer non-Hermitian domain walls, appear in the bandgap. The projected bands of the eight DITISs (Fig.  4d − i) are similar to the bulk bands in Fig. 2e–j. However, the non-Hermitian domain walls cannot result in any new states in the topologically trivial PC (See Fig. 8 of Appendix D).

With OBCs applied in the *x* and *y* directions, the two-dimensional bilayer finite-size C_6v_ topologically nontrivial PC with non-Hermitian domain walls consists of 10 (20) unit cells along the *x* (*y*) direction per layer. The Hamiltonians of the finite-size bilayer non-Hermitian domain walls constructed by nonreciprocal interlayer coupling and on-site gain–loss are ${\widehat{H}}_{\mathrm{C}6-\mathrm{OBC}}^{\mathrm{nonrecip}}$ and ${\widehat{H}}_{\mathrm{C}6-\mathrm{OBC}}^{\mathrm{GL}}$. Figure 4a compares the *E* − *γ* relation given by ${\widehat{H}}_{\mathrm{C}6-\mathrm{OBC}}^{\mathrm{nonrecip}}$ and ${\widehat{H}}_{\mathrm{C}6-\mathrm{OBC}}^{\mathrm{GL}}$. The energy bands are colored according to the ratio ${\rho }_{2}$ of SoFI of the four sites on the boundaries of domain walls to SoFI of all sites. The eigenvalues whose RPs do not vary with *γ* are indicated in blue. The eigenvalues whose RPs vary with *γ* are indicated in purple, which are non-Hermitian DITISs localized at the bilayer non-Hermitian domain walls.

When $\gamma >4.808$, the normalized field distributions of two pairs of the degenerate edge of interface states (EOISs) of ${\widehat{H}}_{\mathrm{C}6-\mathrm{OBC}}^{\mathrm{GL}}$ are localized at the boundaries of non-Hermitian domain walls in the first (second) layer if the IPs of corresponding eigenvalues are positive (negative), and the RPs of corresponding eigenvalues of EOISs are close to zero (Fig. 4b, c). Figure 4d compares the normalized field distributions of two representative EOISs when $\gamma =6$. The normalized field distributions of EOISs of ${\widehat{H}}_{\mathrm{C}6-\mathrm{OBC}}^{\mathrm{nonrecip}}$ are localized at the boundaries of the non-Hermitian domain walls in the first and second layer simultaneously. The transformation matrix between ${\widehat{H}}_{\mathrm{C}6-\mathrm{OBC}}^{\mathrm{nonrecip}}$ and ${\widehat{H}}_{\mathrm{C}6-\mathrm{OBC}}^{\mathrm{GL}}$ is ${\widehat{\mathcal{C}}}_{2400}=\frac{1}{\sqrt{2}}({\widehat{\sigma }}_{x}-{\mathrm{i}\widehat{\sigma }}_{0})\otimes {\widehat{I}}_{1200}$. $|{\varphi }_{\mathrm{C}6-\mathrm{OBC}}^{\mathrm{GL}}\rangle ={\widehat{\mathcal{C}}}_{2400}^{-1}|{\varphi }_{\mathrm{C}6-\mathrm{OBC}}^{\mathrm{nonrecip}}\rangle$ is shown in Fig. 4e, where $|{\varphi }_{\mathrm{C}6-\mathrm{OBC}}^{\mathrm{nonrecip}}\rangle$ and $|{\varphi }_{\mathrm{C}6-\mathrm{OBC}}^{\mathrm{GL}}\rangle$ are the eigenstates of ${\widehat{H}}_{\mathrm{C}6-\mathrm{OBC}}^{\mathrm{nonrecip}}$ and ${\widehat{H}}_{\mathrm{C}6-\mathrm{OBC}}^{\mathrm{GL}}$. The above results indicate that the topological numbers of bilayer non-Hermitian C_6v_-typed DITISs can be defined as is the case for the bilayer non-Hermitian SSH model. The two-dimensional photonic systems can be experimentally realized at microwave frequencies. The photonic crystal platform is based on commercial alumina ceramics (Al_2_O_3_) with bandgap at microwave frequencies [45, 46]. Al_2_O_3_ doped with chromium dioxide can introduce losses [47], so the non-Hermitian control is achieved by doping or not doping chromium with Al_2_O_3_. The non-uniform dissipation distribution can be equivalent to the case of gain–loss distribution [30, 48].

## Conclusion

We have proposed a universal method to equivalently implement nonreciprocal interlayer coupling using on-site gain/loss in one-dimensional and two-dimensional bilayer topological systems through similarity transformation. The similarity transformation provides a convenient tool for understanding and implementing the non-Hermitian skin effect, especially in three-dimensional topological systems. The topological number of the bilayer nonreciprocal interlayer coupling system, which is defined using the gauge-smoothed Wilson loop, can be proved to be equal to the bilayer on-site gain-and-loss system. Topological phase transitions and parity-time-phase transitions of non-Hermitian topological states occur as a result of modulating the strength of nonreciprocal interlayer coupling or on-site gain/loss quantity. The topological origin of DITISs in the C_6v_-typed domain wall can be understood via the bilayer non-Hermitian SSH model because they have the same form of transformation matrices, as is the case for the *E* − *γ* relation and eigenstate characteristics under both PBCs and OBCs. Our results offer new perspectives for studying non-Hermitian topological photonics and manipulating non-Hermitian topological states in bilayer non-Hermitian topological systems. We focused here on a photonic crystal for electromagnetic waves, but a similar lattice design may be applied to other bosonic systems, such as acoustic and mechanical structures [23, 24]. The design principles should be generalizable to various frequencies including radio frequency [49], microwave frequencies [50], and optical frequencies [13].

### Supplementary Information

Below is the link to the electronic supplementary material.

Supplementary file1 (PDF 2475 KB)

## Data Availability

The data that support the findings of this study are available from the corresponding authors upon reasonable request.
