# Supplementary material for: A scheme for realizing nonreciprocal interlayer coupling in bilayer topological systems
Source: Front Optoelectron. 2023 Nov 27;16(1):38. doi: 10.1007/s12200-023-00094-z (PMC10682335; doi:10.1007/s12200-023-00094-z)
Supplement: Supplementary file 1 — Supplementary file1 (PDF 2475 KB) [file 12200_2023_94_MOESM1_ESM.pdf]

Supplemental Materials for

"Equivalent scheme to realize nonreciprocal interlayer coupling in bilayer topological systems"

Xiaoxiao Wang[1], Ruizhe Gu[1], Yandong Li[1], Huixin Qi[1], Xiaoyong Hu[1,2,3,*], Xingyuan Wang [4,†], and Qihuang Gong[1,2,3]

1State Key Laboratory for Mesoscopic Physics & Department of Physics, Collaborative Innovation Center of Quantum Matter & Frontiers Science Center for Nano-optoelectronics, Beijing Academy of Quantum Information Sciences, Peking University, Beijing 100871, P. R. China

2 Peking University Yangtze Delta Institute of Optoelectronics, Nantong, Jiangsu 226010, P. R. China

3 Collaborative Innovation Center of Extreme Optics, Shanxi University, Taiyuan, Shanxi 030006, P. R. China

4 College of Mathematics and Physics, Beijing University of Chemical Technology, Beijing 100029, China

[*]Corresponding authors: xiaoyonghu@ pku.edu.cn

[†]Corresponding authors: wang_xingyuan@mail.buct.edu.cn

APPENDIX A: Bilayer Non-Hermitian SSH Model

Fig. 1(a) in the main text schematically shows the BNH SSH model constructed by stacked photonic waveguide arrays with NIC. The optical field propagation in the waveguide lattice can be described by the coupled-mode theory (CMT) under the tight-binding approximation:

$-\mathrm{i}\frac{\partial}{\partial z}\varphi_n = \beta_0\varphi_n + t_2\varphi_{n-1} + t_1\varphi_{n+1} + (\kappa+\gamma)\varphi_{n+N}$, n=1, 5, 9, …,

$-\mathrm{i}\frac{\partial}{\partial z}\varphi_n = \beta_0\varphi_n + t_1\varphi_{n-1} + t_2\varphi_{n+1} + (\kappa-\gamma)\varphi_{n+N}$, n=2, 6, 10, …,

$-\mathrm{i}\frac{\partial}{\partial z}\varphi_n = \beta_0\varphi_n + t_2\varphi_{n-1} + t_1\varphi_{n+1} + (\kappa-\gamma)\varphi_{n+N}$, n=3, 7, 11, …,

$-\mathrm{i}\frac{\partial}{\partial z}\varphi_n = \beta_0\varphi_n + t_1\varphi_{n-1} + t_2\varphi_{n+1} + (\kappa+\gamma)\varphi_{n+N}$, n=4, 8, 12, …,

$-\mathrm{i}\frac{\partial}{\partial z}\varphi_n = \beta_0\varphi_n + t_2\varphi_{n-1} + t_1\varphi_{n+1} + (\kappa-\gamma)\varphi_{n-N}$, n=N+1, N+5, N+9, …,

$-\mathrm{i}\frac{\partial}{\partial z}\varphi_n = \beta_0\varphi_n + t_1\varphi_{n-1} + t_2\varphi_{n+1} + (\kappa+\gamma)\varphi_{n-N}$, n= N+2, N+6, N+10, …,

$-\mathrm{i}\frac{\partial}{\partial z}\varphi_n = \beta_0\varphi_n + t_2\varphi_{n-1} + t_1\varphi_{n+1} + (\kappa+\gamma)\varphi_{n-N}$, n= N+3, N+7, N+11, …,

$-\mathrm{i}\frac{\partial}{\partial z}\varphi_n = \beta_0\varphi_n + t_1\varphi_{n-1} + t_2\varphi_{n+1} + (\kappa-\gamma)\varphi_{n-N}$, n= N+4, N+8, N+12, …, (A1)

where φ_n denotes the optical field in the *n*th waveguide, β_0 is on-site propagation constants, t_1 and t_2 are two in-layer nearest neighbor coupling coefficients, κ is the isotropic interlayer coupling coefficient, γ

is the anisotropic interlayer coupling coefficient, and N is the number of the waveguides per layer. Fig. 1(b) shows the counterpart with on-site gain and loss, where is no NIC, and its coupled mode equations are given by

$-\mathrm{i}\frac{\partial}{\partial z}\varphi_n = \beta_G\varphi_n + t_2\varphi_{n-1} + t_1\varphi_{n+1} + \kappa\varphi_{n+N}$, n=1, 5, 9, …,

$-\mathrm{i}\frac{\partial}{\partial z}\varphi_n = \beta_L\varphi_n + t_1\varphi_{n-1} + t_2\varphi_{n+1} + \kappa\varphi_{n+N}$, n=2, 6, 10, …,

$-\mathrm{i}\frac{\partial}{\partial z}\varphi_n = \beta_L\varphi_n + t_2\varphi_{n-1} + t_1\varphi_{n+1} + \kappa\varphi_{n+N}$, n=3, 7, 11, …,

$-\mathrm{i}\frac{\partial}{\partial z}\varphi_n = \beta_G\varphi_n + t_1\varphi_{n-1} + t_2\varphi_{n+1} + \kappa\varphi_{n+N}$, n=4, 8, 12, …,

$-\mathrm{i}\frac{\partial}{\partial z}\varphi_n = \beta_L\varphi_n + t_2\varphi_{n-1} + t_1\varphi_{n+1} + \kappa\varphi_{n-N}$, n=N+1, N+5, N+9, …,

$-\mathrm{i}\frac{\partial}{\partial z}\varphi_n = \beta_G\varphi_n + t_1\varphi_{n-1} + t_2\varphi_{n+1} + \kappa\varphi_{n-N}$, n= N+2, N+6, N+10, …,

$-\mathrm{i}\frac{\partial}{\partial z}\varphi_n = \beta_G\varphi_n + t_2\varphi_{n-1} + t_1\varphi_{n+1} + \kappa\varphi_{n-N}$, n= N+3, N+7, N+11, …,

$-\mathrm{i}\frac{\partial}{\partial z}\varphi_n = \beta_L\varphi_n + t_1\varphi_{n-1} + t_2\varphi_{n+1} + \kappa\varphi_{n-N}$, n= N+4, N+8, N+12, …, (A2)

where $\beta_{G,L}$ are the on-site propagation constants ($\beta_G = \beta_0 + i\gamma$ and $\beta_L = \beta_0 - i\gamma$, γ is gain or loss rate) and the other parameters are the same as above. Applying the Fourier transformation, we obtain the Bloch Hamiltonians of the arrays with NIC and on-site gain and loss in the case of PBCs, respectively

$$\hat{H}_{SSH\,PBC}^{nonrecip}(K) = \begin{bmatrix} 0 & t_1 & 0 & t_0e^{iK} & \kappa+\gamma & 0 & 0 & 0 \\ t_1 & 0 & t_0 & 0 & 0 & \kappa-\gamma & 0 & 0 \\ 0 & t_0 & 0 & t_1 & 0 & 0 & \kappa-\gamma & 0 \\ t_0e^{-iK} & 0 & t_1 & 0 & 0 & 0 & 0 & \kappa+\gamma \\ \kappa-\gamma & 0 & 0 & 0 & 0 & t_1 & 0 & t_0e^{iK} \\ 0 & \kappa+\gamma & 0 & 0 & t_1 & 0 & t_0 & 0 \\ 0 & 0 & \kappa+\gamma & 0 & 0 & t_0 & 0 & t_1 \\ 0 & 0 & 0 & \kappa-\gamma & t_0e^{-iK} & 0 & t_1 & 0 \end{bmatrix}, \text{(A3)}$$

which can be viewed as a 2 by 2 block matrix, and it represents the forms of H_m, H_{12}, and H_{21} of Eq. (1).

$$\hat{H}_{SSH\,PBC}^{GL}(K) = \begin{bmatrix} i\gamma & t_1 & 0 & t_2e^{iK} & \kappa & 0 & 0 & 0 \\ t_1 & -i\gamma & t_2 & 0 & 0 & \kappa & 0 & 0 \\ 0 & t_2 & -i\gamma & t_1 & 0 & 0 & \kappa & 0 \\ t_2e^{-iK} & 0 & t_1 & i\gamma & 0 & 0 & 0 & \kappa \\ \kappa & 0 & 0 & 0 & -i\gamma & t_1 & 0 & t_2e^{iK} \\ 0 & \kappa & 0 & 0 & t_1 & i\gamma & t_2 & 0 \\ 0 & 0 & \kappa & 0 & 0 & t_2 & i\gamma & t_1 \\ 0 & 0 & 0 & \kappa & t_2e^{-iK} & 0 & t_1 & -i\gamma \end{bmatrix}. \text{(A4)}$$

The lattice is a bilayer Hermitian SSH model in the absence of the gain and loss ($\gamma = 0$). At the topological phase transition point t_1=t_2, two gapless doubly degenerate Dirac cones at $K = 0$ with energy splitting of

2κ are shown in Fig. 5(b). When $t_1>t_2$ or $t_1<t_2$, two Dirac cones at the center of the FBZ open bandgaps respectively and together form a complete bandgap [Figs. 5(a) and (c)]. The two closest bands above and below the bandgap have a frequency split of 2κ at $K=0$. When $t_1>t_2$, the winding number *ν=0* corresponds to the topologically trivial case, and $t_1<t_2$ corresponds to the topologically nontrivial case with *ν=1*. Eight bands degenerate in pairs at $K=\pm\pi$ creating eight edge DPs, which can be regarded as the spectrum of the original SSH model folded at k = ±π/2 and stretched to the entire FBZ.

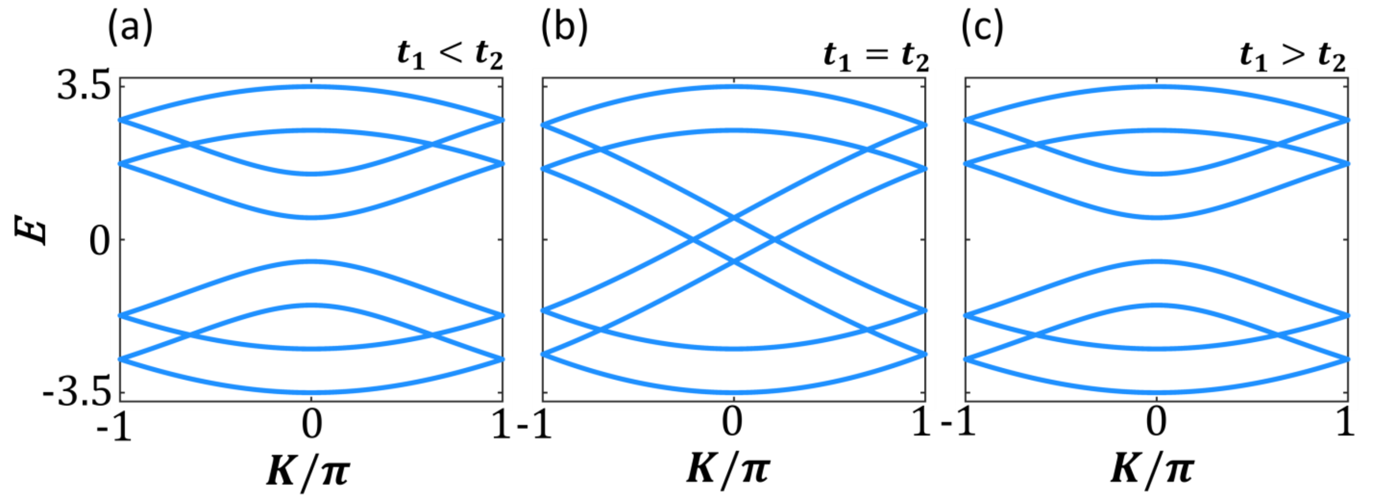

FIG. 5. Folded band structure of bilayer Hermitian SSH model with system parameters κ=0.5, and t_1 =1, $t_2=2$ for (a), $t_1=t_2=1.5$ for (b), and t_1 =2, $t_2=1$ for (c).

Fig. 6 compares the periodic-boundary-condition E−γ relation of $\hat{H}^{nonrecip}_{SSH\ PBC}(K)$ and $\hat{H}^{GL}_{SSH\ PBC}(K)$ when $K=0$, t_1= 2, t_2 = 1, and κ=0.2. Two pairs of EPs emerge at $\gamma=t_1-\kappa$ and $\gamma=t_1+\kappa$, respectively, marked by black dots. Two DPs of the fourth and fifth eigenvalues emerge at $\gamma=\sqrt{(t_1-\kappa)^2-{t_2}^2}$ and $\gamma=\sqrt{(t_1+\kappa)^2-{t_2}^2}$, marked by green dot and red dot, respectively. The DP of the third and the fourth eigenvalues and the DP of the fifth and the sixth eigenvalues emerge at $\gamma=\sqrt{t_1^2+\kappa^2-t_2^2-\frac{t_1^2\kappa^2}{t_2^2}}$, marked by orange dots.

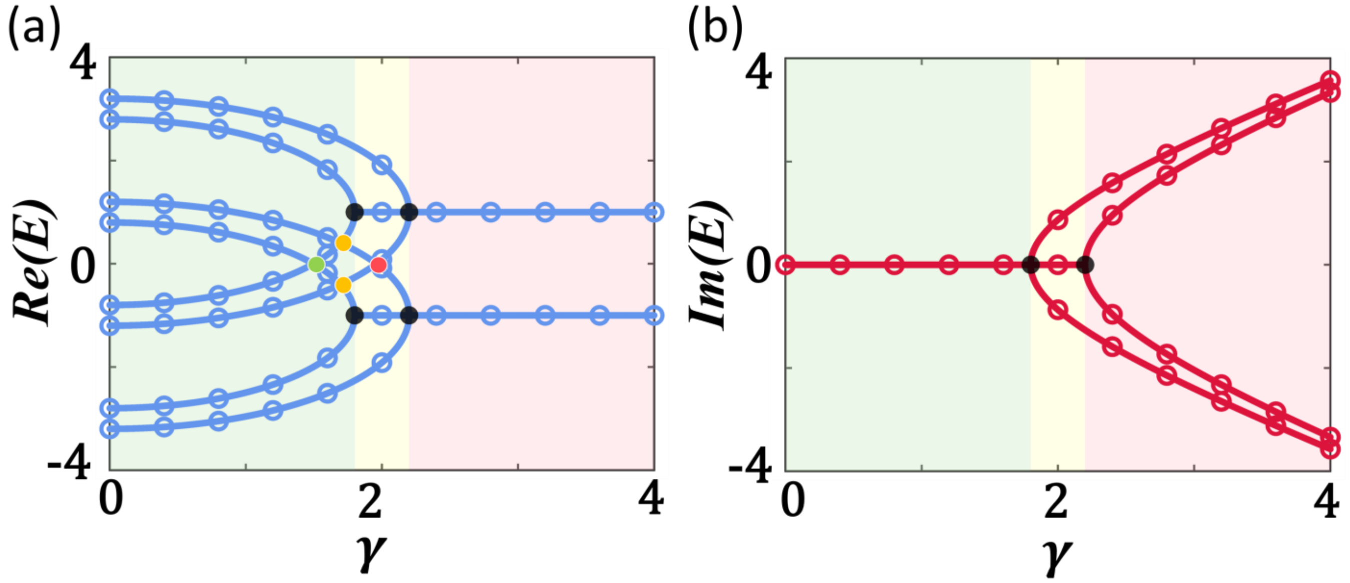

FIG. 6. Comparison of real parts (a) and imaginary parts (b) of the eigenvalues of $\hat{H}^{nonrecip}_{SSH\ PBC}(K)$ (solid lines) and $\hat{H}^{GL}_{SSH\ PBC}(K)$ (discrete circles) as a function of γ. EPs are marked by black dots, and different DPs are marked by green, orange, and red dots.

APPENDIX B: Derivation with Arbitrary Gain and Loss

Considering a two-level system with anisotropic couplings indicated by arrows in various colors, as shown on the left panel of Fig. 7(a). The two-level system with on-site gain and loss is illustrated in the right panel of Fig. 7(a). $\omega_{1,2}$ are the resonance frequencies of two coupled modes, κ denotes the isotropic coupling coefficient, and $\gamma_{1,2}$ are the anisotropic coupling terms in the nonreciprocal hopping model and the gain or loss rate in the on-site gain-and-loss model. By coordinate translation, arbitrary gain and loss can be transformed into a PT-symmetric configuration with a global imaginary part. Without loss of generality, we choose $\gamma_1 = -\gamma_2 = \gamma > 0$, which always holds because we can set the zero point of the imaginary potential as $(\gamma_1 + \gamma_2)/2$. For simplicity, we assume that for the resonance frequencies of two coupled modes, $\omega_1 = \omega_2 = \omega_0$, and that γ, ω_0, and κ are real numbers.

The Hamiltonians of nonreciprocal hopping model and on-site gain-and-loss model using CMT can be written as

$$\hat{H}_2^{nonrecip} = \begin{bmatrix} \omega_0 & \kappa + \gamma \\ \kappa - \gamma & \omega_0 \end{bmatrix}, \text{(B1)}$$

$$\hat{H}_2^{GL} = \begin{bmatrix} \omega_0 + i\gamma & \kappa \\ \kappa & \omega_0 - i\gamma \end{bmatrix}. \text{(B2)}$$

$\hat{H}_2^{GL}$ satisfies PT symmetry $[\hat{H}_2^{GL}, \hat{\mathrm{P}}\hat{\mathrm{T}}] = 0$ with parity operator $\hat{\mathrm{P}}$ and time operator $\hat{\mathrm{T}}$, which act as $\hat{\mathrm{P}}(a_1, a_2) = (a_2, a_1)$ and $\hat{\mathrm{T}}(a_1, a_2) = (a_1{}^*, a_2{}^*)$. $\hat{H}_2^{GL}$ has PT symmetry since $(\hat{\mathrm{P}}\hat{\mathrm{T}})\hat{H}_2^{GL}(\hat{\mathrm{P}}\hat{\mathrm{T}})^{-1} = \hat{H}_2^{GL}$,

where $\hat{\mathrm{P}} = \hat{\sigma}_x$ and $\hat{\mathrm{T}}$ is the complex-conjugation operation. The values of the parameters in $\hat{H}_2^{nonrecip}$ and $\hat{H}_2^{GL}$ are the same. The eigenvalues of both systems are $\mathrm{E}_2^{nonrecip} = E_2^{GL} = \omega_0 \pm \sqrt{\kappa^2 - \gamma^2}$. In Fig. 7(b), we compare the behavior of the eigenvalues of $\hat{H}_2^{nonrecip}$ and $\hat{H}_2^{GL}$ as γ changes when $\kappa = 1$ and $\omega_0 = 0$.

The similarity transformation is still applicable when the zero point of the imaginary potential is not equal to 0. Arbitrary gain and loss (γ_1 and γ_2, both can be positive and negative) are introduced in the on-site gain-and-loss two-level system. The Hamiltonian can be written as

$$H_2^{GL_{arb}} = \begin{pmatrix} \omega_0 + i\gamma_1 & \kappa \\ \kappa & \omega_0 + i\gamma_2 \end{pmatrix}. \text{ (B3)}$$

$\xi = (\gamma_1 + \gamma_2)/2$ and $\eta = (\gamma_1 - \gamma_2)/2$ are introduced, since arbitrary gain and loss can be seen as the coordinate translation of PT-symmetric structure, and the imaginary part of the eigenvalues will get a corresponding shift.

The Hamiltonian in Eq. (B3) is transformed to PT-symmetric two-level system:

$$H_2^{GL'} = \begin{pmatrix} \omega_0 + i\eta & \kappa \\ \kappa & \omega_0 - i\eta \end{pmatrix}, \text{ with a uniform background } \Delta H_2^{GL} = \begin{pmatrix} i\xi & 0 \\ 0 & i\xi \end{pmatrix}. \text{ (B4)}$$

The Hamiltonian of nonreciprocal two-level system is

$$H_2^{nonrecip'} = \begin{pmatrix} \omega_0 & \kappa + \eta \\ \kappa - \eta & \omega_0 \end{pmatrix}, \text{ with a reciprocal coupling } \Delta H_2^{nonrecip} = \begin{pmatrix} 0 & \xi \\ \xi & 0 \end{pmatrix}. \text{ (B5)}$$

The similarity transformation relates $H_2^{GL'}$ and $H_2^{nonrecip'}$

$$\hat{C}_2 H_2^{GL'} \hat{C}_2^{-1} - H_2^{nonrecip'} = 0,\ \hat{C}_2 = \frac{1}{\sqrt{2}} \begin{pmatrix} -i & 1 \\ 1 & -i \end{pmatrix}. \text{ (B6)}$$

Four-level systems with NIC and balanced on-site gain and loss are shown in Fig. 7(c). t is the in-layer hopping strength and we normalize all quantities using t. $\omega_{1,2,3,4}$ are the resonance frequencies of four coupled modes. κ denotes the isotropic interlayer coupling coefficient. $\gamma_{1,2,3,4}$ are the anisotropic interlayer coupling terms in the nonreciprocal hopping model and the gain or loss rate in the on-site gain-and-loss model. We choose $\gamma_1 = -\gamma_3 = -\gamma_2 = \gamma_4 = \gamma > 0$. Using CMT, the Hamiltonians are written as follows:

$$\hat{H}_4^{nonrecip} = t\hat{\sigma}_0 \otimes \hat{\sigma}_x + \kappa \hat{\sigma}_x \otimes \hat{\sigma}_0 + i\gamma \hat{\sigma}_y \otimes \hat{\sigma}_z, \text{ (B7)}$$

$$\hat{H}_4^{GL} = t\hat{\sigma}_0 \otimes \hat{\sigma}_x + \kappa \hat{\sigma}_x \otimes \hat{\sigma}_0 + i\gamma \hat{\sigma}_z \otimes \hat{\sigma}_z, \text{ (B8)}$$

where $\hat{\sigma}_0$ and $\hat{\sigma}_{x,y,z}$ are the two-by-two identity matrix and Pauli matrix. $\hat{H}_4^{GL}$ has the anti-PT symmetry $(\hat{\mathrm{P}}\hat{\mathrm{T}})\hat{H}_4^{GL}(\hat{\mathrm{P}}\hat{\mathrm{T}})^{-1} = -\hat{H}_4^{GL}$ with $\hat{\mathrm{P}} = i\hat{\sigma}_x \otimes \hat{\sigma}_y$ and complex-conjugation operation $\hat{\mathrm{T}}$. A Hamiltonian $\hat{\mathrm{H}}$ is anti-PT-symmetric when $\{\hat{\mathrm{H}}, \hat{\mathrm{P}}\hat{\mathrm{T}}\} = 0$, where the commutator replaced by the anticommutator, which represents a generalization of PT symmetry. We obtain the equal eigenvalues of Eqs. (B7) and (B8) as

$E_4^{nonrecip} = E_4^{GL} = \omega_0 \pm \sqrt{(\kappa \pm t)^2 - \gamma^2}$. In Fig. 7(d), we compare the $E-\gamma$ relation of the four eigenvalues of $\hat{H}_4^{nonrecip}$ (solid lines) and $\hat{H}_4^{GL}$ (discrete circles) when $\kappa/t = 2$ and $\omega_0 = 0$. Two EPs emerge at $\gamma = |\kappa - t|$ and $\gamma = |\kappa + t|$, respectively, indicated by black dots.

Arbitrary gain and loss can also be introduced in the on-site gain-and-loss four-level system. The Hamiltonian can be written as

$$H_4^{GL_{arb}} = \begin{pmatrix} \omega_0 + i\gamma_1 & t & \kappa & 0 \\ t & \omega_0 + i\gamma_2 & 0 & \kappa \\ \kappa & 0 & \omega_0 + i\gamma_3 & t \\ 0 & \kappa & t & \omega_0 + i\gamma_4 \end{pmatrix}, \text{(B9)}$$

$\xi_1 = (\gamma_1 + \gamma_3)/2$, $\eta_1 = (\gamma_1 - \gamma_3)/2$, $\xi_2 = (\gamma_4 + \gamma_2)/2$, and $\eta_2 = (\gamma_4 - \gamma_2)/2$ are introduced, since arbitrary gain and loss can be seen as the coordinate translation of PT-symmetric structure, and the imaginary part of the eigenvalues will get a corresponding shift.

The Hamiltonian in Eq. (B9) is transformed to a PT-symmetric four-level system: $H_4^{GL'} = \begin{pmatrix} \omega_0 + i\eta_1 & t & \kappa & 0 \\ t & \omega_0 - i\eta_2 & 0 & \kappa \\ \kappa & 0 & \omega_0 - i\eta_1 & t \\ 0 & \kappa & t & \omega_0 + i\eta_2 \end{pmatrix}$, with background $\Delta H_4^{GL} = \begin{pmatrix} i\xi_1 & 0 & 0 & 0 \\ 0 & i\xi_2 & 0 & 0 \\ 0 & 0 & i\xi_1 & 0 \\ 0 & 0 & 0 & i\xi_2 \end{pmatrix}$. (B10)

The Hamiltonian of nonreciprocal four-level system is $H_4^{nonrecip'} = \begin{pmatrix} \omega_0 & t & \kappa + \eta_1 & 0 \\ t & \omega_0 & 0 & \kappa - \eta_2 \\ \kappa - \eta_1 & 0 & \omega_0 & t \\ 0 & \kappa + \eta_2 & t & \omega_0 \end{pmatrix}$,

$$\text{with reciprocal coupling } \Delta H_4^{nonrecip} = \begin{pmatrix} 0 & 0 & \xi_1 & 0 \\ 0 & 0 & 0 & \xi_2 \\ \xi_1 & 0 & 0 & 0 \\ 0 & \xi_2 & 0 & 0 \end{pmatrix}. \text{(B11)}$$

The similarity transformation relates $H_4^{GL'}$ and $H_4^{nonrecip'}$

$$\hat{C}_4 H_4^{GL'} \hat{C}_4^{-1} - H_4^{nonrecip'} = 0,\ \hat{C}_4 = \frac{1}{\sqrt{2}} \begin{pmatrix} -i & 0 & 1 & 0 \\ 0 & -i & 0 & 1 \\ 1 & 0 & -i & 0 \\ 0 & 1 & 0 & -i \end{pmatrix}. \text{(B12)}$$

The coordinate translation can also be used to transform arbitrary gain and loss in BNH SSH model, and BNH C_{6v} PC.

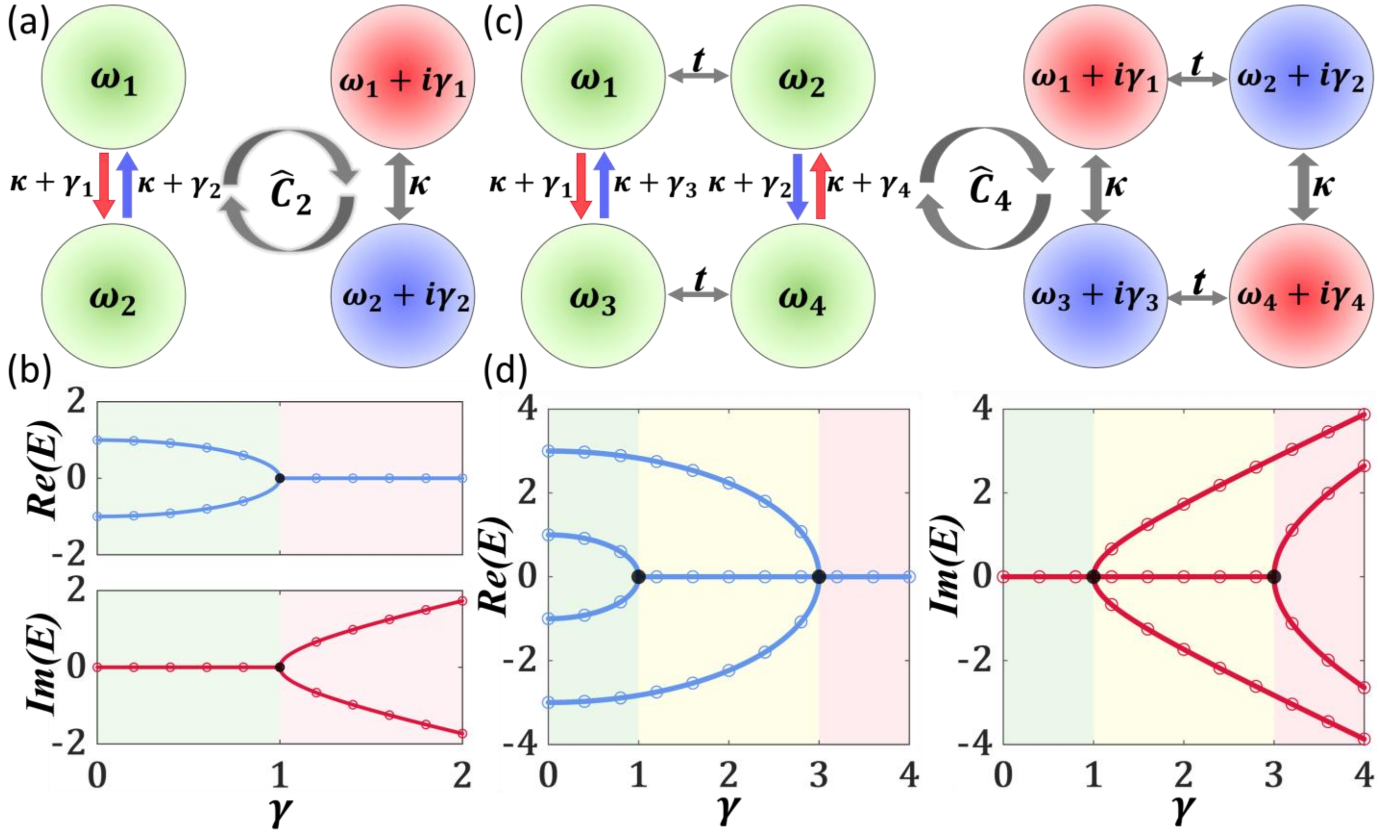

FIG. 7. (a) Schematic diagram of two non-Hermitian two-level systems with nonreciprocal coupling and balanced gain and loss, whose Hamiltonians have the same eigenvalues and satisfy $\hat{C}_2\hat{H}_2^{GL}\hat{C}_2^{-1} = \hat{H}_2^{nonrecip}$. (b) Comparison of the energy spectrum given by $\hat{H}_2^{nonrecip}$ (solid lines) and $\hat{H}_2^{GL}$ (discrete circles) as a function of γ. (c) Schematic diagram of two non-Hermitian four-level systems with nonreciprocal coupling and balanced gain and loss, whose Hamiltonians have the same eigenvalues and satisfy $\hat{C}_4\hat{H}_4^{GL}\hat{C}_4^{-1} = \hat{H}_4^{nonrecip}$. (d) Comparison of the energy spectrum given by $\hat{H}_4^{nonrecip}$ (solid lines) and $\hat{H}_4^{GL}$ (discrete circles) as a function of γ.

A PT-symmetric trimer system with gain and loss γ on the outer matrix entries and only a frequency offset Δ on the center entry is considered now, described by the Hamiltonian:

$$\hat{H}_3^{PT} = \begin{pmatrix} i\gamma & t & 0 \\ t & \Delta & t \\ 0 & t & -i\gamma \end{pmatrix}. \text{(B13)}$$

The bilayer PT-symmetric trimer system is shown in the left panel of Fig. 8(a), and the bilayer trimer system with NIC is shown in the right panel of Fig. 8(a). The Hamiltonians of the bilayer PT-symmetric trimer system and the bilayer nonreciprocal trimer system are

$$\hat{H}_6^{GL} = \begin{pmatrix} i\gamma & t & 0 & \kappa & 0 & 0 \\ t & \Delta & t & 0 & \kappa & 0 \\ 0 & t & -i\gamma & 0 & 0 & \kappa \\ \kappa & 0 & 0 & -i\gamma & t & 0 \\ 0 & \kappa & 0 & t & \Delta & t \\ 0 & 0 & \kappa & 0 & t & i\gamma \end{pmatrix}, \text{ and } \hat{H}_6^{nonrecip} = \begin{pmatrix} 0 & t & 0 & \kappa+\gamma & 0 & 0 \\ t & \Delta & t & 0 & \kappa & 0 \\ 0 & t & 0 & 0 & 0 & \kappa-\gamma \\ \kappa-\gamma & 0 & 0 & 0 & t & 0 \\ 0 & \kappa & 0 & t & \Delta & t \\ 0 & 0 & \kappa+\gamma & 0 & t & 0 \end{pmatrix}. \text{(B14)}$$

The similarity transformation relates $\hat{H}_6^{GL}$ and $\hat{H}_6^{nonrecip}$ are

$$\hat{C}_6 \hat{H}_6^{GL} \hat{C}_6^{-1} = \hat{H}_6^{nonrecip}, \text{ with } \hat{C}_6 = \frac{1}{\sqrt{2}}(\hat{\sigma}_x - i\hat{\sigma}_0) \otimes \hat{I}_3. \text{ (B15)}$$

In Fig. 8(b), we compare the $E-\gamma$ relation of the six eigenvalues of $\hat{H}_6^{nonrecip}$ (solid lines) and $\hat{H}_6^{GL}$ (discrete circles) when $t = 1$, $\kappa = 2$, and $\Delta = 1$. Two EPs are indicated by black dots.

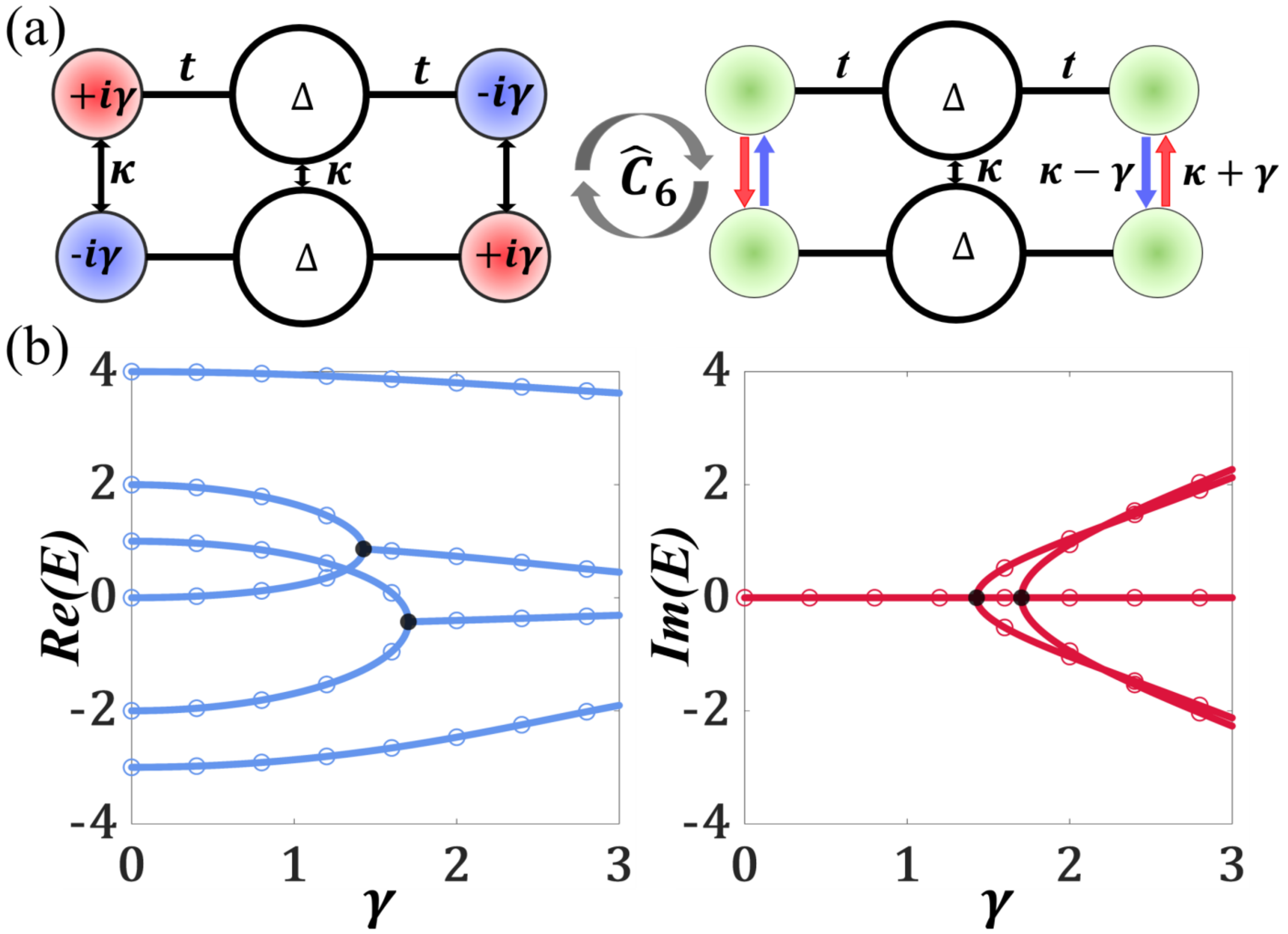

FIG. 8 (a) Schematic diagram of bilayer PT-symmetric trimer system and the bilayer trimer system with anisotropic interlayer coupling. (b) Comparison of the energy spectrum given by $\hat{H}_6^{nonrecip}$ (solid lines) and $\hat{H}_6^{GL}$ (discrete circles) in (a) as a function of γ.

APPENDIX C: Properties of Similarity Transformation

The more general forms of the Hamiltonians of the systems with NIC and on-site gain/loss are given by

$$\hat{H}_n^{nonrecip} = \begin{bmatrix} \hat{H}_0 & \hat{G} - \mathrm{i}\hat{D} \\ \hat{G} + \mathrm{i}\hat{D} & \hat{H}_0 \end{bmatrix}, \text{(C1)}$$

$$\hat{H}_n^{GL} = \begin{bmatrix} \hat{H}_0 + \hat{D} & \hat{G} \\ \hat{G} & \hat{H}_0 - \hat{D} \end{bmatrix}, \text{(C2)}$$

Both Hamiltonians are written as 2×2 block matrices, $\hat{\mathrm{H}}_0$ is a Hermitian matrix describing on-site energy and in-layer isotropic hopping, $\hat{G}$ is a diagonal matrix describing isotropic interlayer hopping. $\hat{D}$ is a diagonal matrix describing anisotropic interlayer hopping in $\hat{H}_n^{nonrecip}$, and describes on-site gain and loss in $\hat{H}_n^{GL}$. The similarity transformation is described as $\hat{C}_n \hat{H}_n^{GL} \hat{C}_n^{-1} = \hat{H}_n^{nonrecip}$ and its Hermitian conjugate form $\hat{C}_n {\hat{H}_n^{GL}}^{\dagger} \hat{C}_n^{-1} = {\hat{H}_n^{nonrecip}}^{\dagger}$, which brings $\hat{D}$ from diagonal to off-diagonal, which means we convert the system with nonreciprocal coupling into the system with on-site gain and loss. The eigenvalues of the systems with on-site gain\loss and nonreciprocal hopping are the same, and the eigenstates belong to the same eigenvalues of two Hamiltonians are related by $\hat{C}_n$.

The eigenequations of $\hat{H}_n^{GL}$ and $\hat{H}_n^{nonrecip}$ are

$$\hat{H}_n^{GL} |\phi_m^{GL}\rangle = E_m |\phi_m^{GL}\rangle, \text{ and } {\hat{H}_n^{GL}}^{\dagger} |\chi_m^{GL}\rangle = E_m^* |\chi_m^{GL}\rangle, \text{(C3)}$$

$$\hat{H}_n^{nonrecip} |\phi_m^{nonrecip}\rangle = E_m |\phi_m^{nonrecip}\rangle, \text{ and } {\hat{H}_n^{nonrecip}}^{\dagger} |\chi_m^{nonrecip}\rangle = E_m^* |\chi_m^{nonrecip}\rangle. \text{(C4)}$$

where $|\phi_m^{GL}\rangle$ and $|\chi_m^{GL}\rangle$ ($|\phi_m^{nonrecip}\rangle$ and $|\chi_m^{nonrecip}\rangle$) are the mth right and left eigenstates of $\hat{H}_n^{GL}(K_i)$ ($\hat{H}_n^{nonrecip}$).

By using the similarity transformation, we can obtain

$$\hat{C}_n \hat{H}_n^{GL} \hat{C}_n^{-1} |\phi_m^{nonrecip}\rangle = E_m |\phi_m^{nonrecip}\rangle, \text{(C5)}$$

left multiply Eq. (C5) by $\hat{C}_n^{-1}$, and we get $\hat{H}_n^{GL} \hat{C}_n^{-1} |\phi_m^{nonrecip}\rangle = E_m \hat{C}_n^{-1} |\phi_m^{nonrecip}\rangle$, which means $\hat{C}_n^{-1} |\phi_m^{nonrecip}\rangle$ is an eigenstate of $\hat{H}_n^{GL}$.

Another form of the similarity transformation is $\hat{H}_n^{GL} = \hat{C}_n^{-1} \hat{H}_n^{nonrecip} \hat{C}_n$, and we can obtain $\hat{C}_n^{-1} \hat{H}_n^{nonrecip} \hat{C}_n |\phi_m^{GL}\rangle = E_m |\phi_m^{GL}\rangle$. Multiplying the above equation left by $\hat{C}_n$, and we get $\hat{H}_n^{nonrecip} \hat{C}_n |\phi_m^{GL}\rangle = E_m \hat{C}_n |\phi_m^{GL}\rangle$), which means $\hat{C}_n |\phi_m^{GL}\rangle$ is an eigenstate of $\hat{H}_n^{nonrecip}$. The relations of eigenstates are $|\phi_m^{GL}\rangle = \hat{C}_n^{-1} |\phi_m^{nonrecip}\rangle$ and $|\phi_m^{nonrecip}\rangle = \hat{C}_n |\phi_m^{GL}\rangle$. In the same way, the *m*th left

eigenstates belonging to the same eigenvalues of the two Hamiltonians ${\hat{H}_n^{nonrecip}}^\dagger$ and ${\hat{H}_n^{GL}}^\dagger$ are related by the transformation matrix $\hat{C}_n$:

$$|\chi_m^{GL}\rangle = \hat{C}_n^{-1}|\chi_m^{nonrecip}\rangle \text{ and } |\chi_m^{nonrecip}\rangle = \hat{C}_n|\chi_m^{GL}\rangle. \text{ (C6)}$$

The similarity transformation can be understood as an operation on the basis set where the Hamiltonians are written. The tight-binding Hamiltonians are originally given based on wannier states, for example, the lattice sites. However, consider the linear combination of the two corresponding bases with a phase of $\frac{\pi}{2}$ or $-\frac{\pi}{2}$ to form a new set of bases, and we find the transformation matrix is exactly $\hat{C}_n^{-1}$. As a result, the system with nonreciprocal hopping and the system with on-site gain and loss may be regarded as alternative expressions of the same non-Hermitian system.

APPENDIX D: Bilayer Domain Walls of C_{6V} PC

The matrix forms of H_{mono}, H_{GL}, and H_{LG} in Eq. (12) are as follows:

$$H_{mono} = \begin{bmatrix} \hat{H}_0 & \hat{H}_{c1} & 0 & 0 & 0 & 0 & 0 \\ \hat{H}_{c2} & \hat{H}_0 & \hat{H}_{c1} & 0 & 0 & 0 & 0 \\ 0 & \hat{H}_{c2} & \hat{H}_0 & \hat{H}_{c1} & 0 & 0 & 0 \\ 0 & 0 & \hat{H}_{c2} & \ddots & \hat{H}_{c1} & 0 & 0 \\ 0 & 0 & 0 & \hat{H}_{c2} & \hat{H}_0 & \hat{H}_{c1} & 0 \\ 0 & 0 & 0 & 0 & \hat{H}_{c2} & \hat{H}_0 & \hat{H}_{c1} \\ 0 & 0 & 0 & 0 & 0 & \hat{H}_{c2} & \hat{H}_0 \end{bmatrix}, \text{ (D1)}$$

$$H_{GL} = \begin{bmatrix} \hat{H}_G & \hat{H}_{c1} & 0 & 0 & 0 & 0 & 0 \\ \hat{H}_{c2} & \hat{H}_G & \hat{H}_{c1} & 0 & 0 & 0 & 0 \\ 0 & \hat{H}_{c2} & \hat{H}_G & \hat{H}_{c1} & 0 & 0 & 0 \\ 0 & 0 & \hat{H}_{c2} & \ddots & \hat{H}_{c1} & 0 & 0 \\ 0 & 0 & 0 & \hat{H}_{c2} & \hat{H}_L & \hat{H}_{c1} & 0 \\ 0 & 0 & 0 & 0 & \hat{H}_{c2} & \hat{H}_L & \hat{H}_{c1} \\ 0 & 0 & 0 & 0 & 0 & \hat{H}_{c2} & \hat{H}_l \end{bmatrix}, \text{ (D2)}$$

$$H_{LG} = \begin{bmatrix} \hat{H}_L & \hat{H}_{c1} & 0 & 0 & 0 & 0 & 0 \\ \hat{H}_{c2} & \hat{H}_L & \hat{H}_{c1} & 0 & 0 & 0 & 0 \\ 0 & \hat{H}_{c2} & \hat{H}_L & \hat{H}_{c1} & 0 & 0 & 0 \\ 0 & 0 & \hat{H}_{c2} & \ddots & \hat{H}_{c1} & 0 & 0 \\ 0 & 0 & 0 & \hat{H}_{c2} & \hat{H}_G & \hat{H}_{c1} & 0 \\ 0 & 0 & 0 & 0 & \hat{H}_{c2} & \hat{H}_G & \hat{H}_{c1} \\ 0 & 0 & 0 & 0 & 0 & \hat{H}_{c2} & \hat{H}_G \end{bmatrix}, \text{ (D3)}$$

where the matrices $\hat{H}_G$, $\hat{H}_L$, $\hat{H}_{c1}$, and $\hat{H}_{c2}$ take the forms of

$$\widehat{H}_{G/L}=\begin{bmatrix}\pm i\gamma & t_0 & 0 & 0 & 0 & t_0 & 0 & 0 & 0 & 0 & 0 & 0\\ t_0 & \pm i\gamma & t_0 & 0 & te^{iK} & 0 & 0 & 0 & 0 & 0 & 0 & 0\\ 0 & t_0 & \pm i\gamma & t_0 & 0 & 0 & t & 0 & 0 & 0 & 0 & 0\\ 0 & 0 & t_0 & \pm i\gamma & t_0 & 0 & 0 & 0 & 0 & t & 0 & 0\\ 0 & te^{-iK} & 0 & t_0 & \pm i\gamma & t_0 & 0 & 0 & 0 & 0 & 0 & 0\\ t_0 & 0 & 0 & 0 & t_0 & \pm i\gamma & 0 & 0 & 0 & 0 & 0 & 0\\ 0 & 0 & t & 0 & 0 & 0 & \pm i\gamma & t_0 & 0 & t_0e^{iK} & 0 & 0\\ 0 & 0 & 0 & 0 & 0 & 0 & t_0 & \pm i\gamma & t_0 & 0 & t & 0\\ 0 & 0 & 0 & 0 & 0 & 0 & 0 & t_0 & \pm i\gamma & 0 & 0 & t_0e^{iK}\\ 0 & 0 & 0 & t & 0 & 0 & t_0e^{-iK} & 0 & 0 & \pm i\gamma & t_0 & 0\\ 0 & 0 & 0 & 0 & 0 & 0 & 0 & t & 0 & t_0 & \pm i\gamma & t_0\\ 0 & 0 & 0 & 0 & 0 & 0 & 0 & 0 & t_0e^{-iK} & 0 & t_0 & \pm i\gamma\end{bmatrix}, \text{(D4)}$$

$$\widehat{H}_{c1}=\begin{bmatrix}0&0&0&0&0&0&0&0&0&0&0&0\\0&0&0&0&0&0&0&0&0&0&0&0\\0&0&0&0&0&0&0&0&0&0&0&0\\0&0&0&0&0&0&0&0&0&0&0&0\\0&0&0&0&0&0&0&0&0&0&0&0\\0&0&0&0&0&0&0&0&0&0&0&0\\0&0&0&0&0&0&0&0&0&0&0&0\\0&0&0&0&0&0&0&0&0&0&0&0\\t&0&0&0&0&0&0&0&0&0&0&0\\0&0&0&0&0&0&0&0&0&0&0&0\\0&0&0&0&0&0&0&0&0&0&0&0\\0&0&0&0&0&t&0&0&0&0&0&0\end{bmatrix}, \text{(D5)}$$

$$\widehat{H}_{c2}=\begin{bmatrix}0&0&0&0&0&0&0&0&t&0&0&0\\0&0&0&0&0&0&0&0&0&0&0&0\\0&0&0&0&0&0&0&0&0&0&0&0\\0&0&0&0&0&0&0&0&0&0&0&0\\0&0&0&0&0&0&0&0&0&0&0&0\\0&0&0&0&0&0&0&0&0&0&0&t\\0&0&0&0&0&0&0&0&0&0&0&0\\0&0&0&0&0&0&0&0&0&0&0&0\\0&0&0&0&0&0&0&0&0&0&0&0\\0&0&0&0&0&0&0&0&0&0&0&0\\0&0&0&0&0&0&0&0&0&0&0&0\\0&0&0&0&0&0&0&0&0&0&0&0\end{bmatrix}, \text{(D6)}$$

The subscripts of $\widehat{H}_{G/L}$ represent on-site gain (diagonal element of the $\widehat{H}_G$ is $+i\gamma$) or loss (diagonal element of the $\widehat{H}_L$ is $-i\gamma$). $\widehat{H}_0$ equals $\widehat{H}_{G/L}$ when $\gamma = 0$. $\widehat{H}_{c1}$ and $\widehat{H}_{c2}$ represent the coupling between this unit and its two neighboring units, respectively. The first and last units of both layers can exchange energy with the environment for the scattering boundary conditions, so they only couple with one neighboring unit.

Fig. 9(a) is the zoom-out plot of Fig. 3(i), showing the complete projected band structures in the topological case. The domain-induced bands are colored in blue (real parts) and red (imaginary parts). Fig. 9(b) compares the projected band structures given by $\widehat{H}^{nonrecip}_{C6\,PBC}(K)$ (solid lines) and $\widehat{H}^{GL}_{C6\,PBC}(K)$ (discrete circles) in the

trivial case with parameters t_1= 0.5, $t_2 = 1$, κ=0.05, and γ = 4.934. In the topological case, eight bands appear in the bandgap because C6v-typed cross-shaped gain-loss domain walls can support localized interface states protected by the corresponding Hermitian topology. However, the domain wall cannot result in any new states in the trivial case, because no modes appear at the boundaries of a subsystem in the trivial cases.

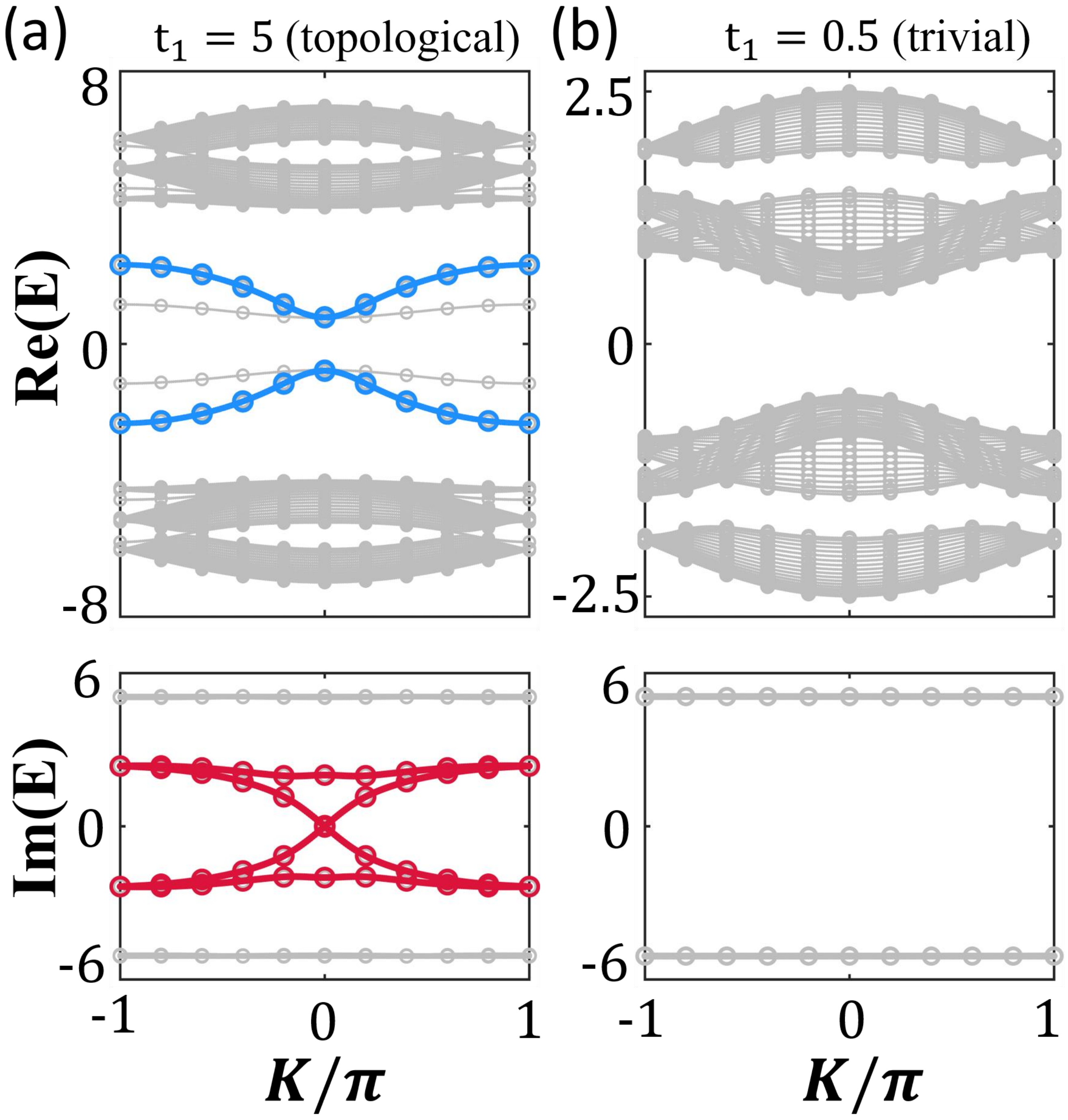

FIG. 9. Comparison of projected bands given by $\hat{H}^{nonrecip}_{C6\,PBC}(K)$ (solid lines) and $\hat{H}^{GL}_{C6\,PBC}(K)$ (discrete circles) with parameters γ = 4.934, $t_2 = 1$, $\kappa = 0.05$, and $t_1 = 5$ for (a) and t_1= 0.5 for (b).

The zoom-in plot of Fig. 3(c) is shown in the Fig. 10(a), where the DITISs with different PT symmetry–

breaking threshold points are colored in dark and light blue (red) in real (imaginary) parts. In Fig. 10(b), we compare the normalized field distributions of four representative DITISs of $\hat{H}^{nonrecip}_{C6\,PBC}(K)$ (light green) and $\hat{H}^{GL}_{C6\,PBC}(K)$ (dark green) when γ = 4.87. The normalized field distribution of DITISs of $\hat{H}^{GL}_{C6\,PBC}(K)$ is uniformly distributed in the four regions (colored by light pink and blue) in exact PT symmetry phase, and mainly distributed in the crossed regions colored pink (blue) if the imaginary parts of corresponding eigenvalues are positive (negative) in broken PT symmetry phase.$|\varphi^{GL}_{C6\,PBC}\rangle = \hat{C}^{-1}_{480}|\varphi^{nonrecip}_{C6\,PBC}\rangle$ is shown in the Fig. 10(c), where $|\varphi^{nonrecip}_{C6\,PBC}\rangle$ ($|\varphi^{GL}_{C6\,PBC}\rangle$) are the eigenstates of $\hat{H}^{nonrecip}_{C6\,PBC}(K)$ ($\hat{H}^{GL}_{C6\,PBC}(K)$).

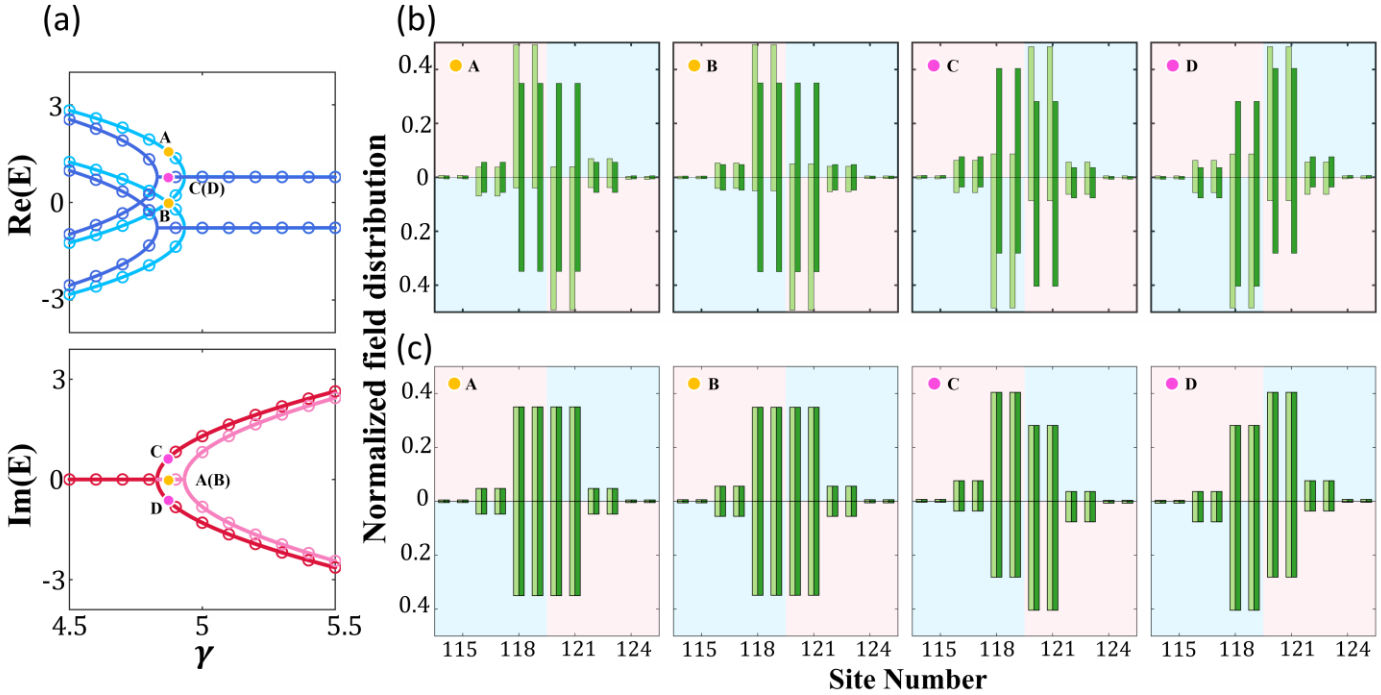

FIG. 10. (a) Comparison of the E−γ relation of DITISs of $\hat{H}^{nonrecip}_{C6\,PBC}(K)$ (solid line) and $\hat{H}^{GL}_{C6\,PBC}(K)$ (discrete circles) at $K = 0$. (b) Comparison of $|\varphi^{nonrecip}_{C6\,PBC}\rangle$ (light green) and $|\varphi^{GL}_{C6\,PBC}\rangle$ (dark green) for the four representative DITISs. (c) Comparison of $\hat{C}^{-1}_{480}|\varphi^{nonrecip}_{C6\,PBC}\rangle$ (light green) and $|\varphi^{GL}_{C6\,PBC}\rangle$ (dark green) for the four representative DITISs. Above (below) the horizontal line is the field distribution of the first (second) layer. Only twelve sites around the domain wall of both layers are shown.

APPENDIX E: Type2 Similarity Transformation

We present another similarity transformation between the non-Hermitian systems with nonreciprocal coupling and on-site gain/loss. Considering another kind of four-level system with nonreciprocal coupling and its counterpart with balanced on-site gain/loss [Fig. 11(a)]. By using CMT, the Hamiltonians are written as:

$$\hat{H}_{4'}^{nonrecip} = \begin{bmatrix} \omega_0 & t & \kappa + i\gamma & 0 \\ t & \omega_0 & 0 & \kappa - i\gamma \\ \kappa + i\gamma & 0 & \omega_0 & t \\ 0 & \kappa - i\gamma & t & \omega_0 \end{bmatrix}, \text{(E1)}$$

$$\hat{H}_{4'}^{GL} = \begin{bmatrix} \omega_0 + i\gamma & t & \kappa & 0 \\ t & \omega_0 - i\gamma & 0 & \kappa \\ \kappa & 0 & \omega_0 + i\gamma & t \\ 0 & \kappa & t & \omega_0 - i\gamma \end{bmatrix}, \text{(E2)}$$

where ω_0, t, κ, and γ are real numbers. ω_0 is the frequency of the four coupled modes since $\omega_1 = \omega_2 = \omega_3 = \omega_4 = \omega_0$, t is the in-layer hopping strength, and we normalize all quantities with t. κ represents the isotropic coupling coefficient, and γ represents the non-Hermitian part of interlayer hopping in Eq. (E1) and the gain or loss rate of the coupled modes in Eq. (E2), respectively. We obtain the equal eigenvalues of $\hat{H}_{4'}^{nonrecip}$ and $\hat{H}_{4'}^{GL}$ as $\mathrm{E}_{4'}^{nonrecip} = E_{4'}^{GL} = \omega_0 \pm \kappa \pm \sqrt{t^2 - \gamma^2}$. Fig. 11(b) compares the E – γ relation of the four eigenvalues of $\hat{H}_{4'}^{nonrecip}$ (solid lines) and $\hat{H}_{4'}^{GL}$ (discrete circles) when *κ/t=2* and $\omega_0 = 0$. Two EPs emerge simultaneously at $\gamma = t$, marked by black dots. The type2 transformation matrix is

$$\hat{C}_{4'} = \begin{bmatrix} 1 & -i\gamma & 0 & i\gamma \\ 0 & 1 & 0 & 0 \\ 0 & i\gamma & 1 & -i\gamma \\ 0 & 0 & 0 & 1 \end{bmatrix}, \text{(E3)}$$

The type2 similarity transformation is defined as $\hat{C}_{4'}\hat{H}_{4'}^{GL}\hat{C}_{4'}^{-1} = \hat{H}_{4'}^{nonrecip}$, and the relation of their right eigenstates satisfy $|\phi_m^{GL}\rangle = \hat{C}_{4'}^{-1}|\phi_m^{nonrecip}\rangle$, where $\hat{C}_{4'}^{-1} = \begin{bmatrix} 1 & i\gamma & 0 & -i\gamma \\ 0 & 1 & 0 & 0 \\ 0 & -i\gamma & 1 & i\gamma \\ 0 & 0 & 0 & 1 \end{bmatrix}$.

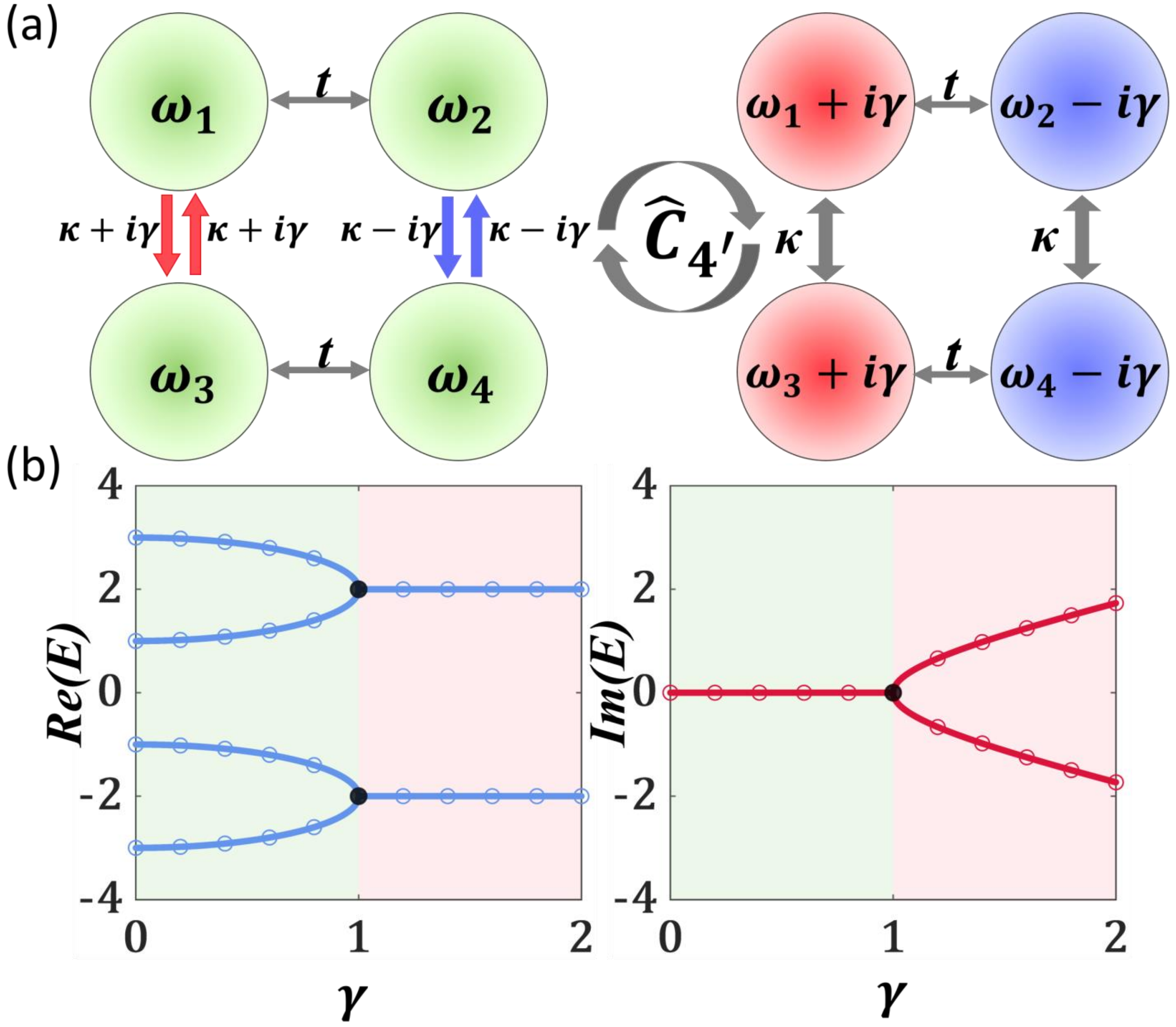

FIG. 11. (a) Schematic diagram of two non-Hermitian four-level systems with nonreciprocal coupling and balanced gain and loss, whose Hamiltonians have the same eigenvalues and satisfy the type2 similarity transformation. (b) Comparison of the energy spectrum given by $\hat{H}_{4'}^{nonrecip}$ (solid lines) and $\hat{H}_{4'}^{GL}$ (discrete circles) as a function of γ.

Consider the BNH SSH model constructed by stacked photonic waveguide arrays in Figs. 12(a) and (b), whose non-Hermiticity is caused by NIC and on-site gain/loss, respectively. Following the CMT in the tight-binding approximation and applying the Fourier transformation, we obtain the Bloch Hamiltonians of the unit cellin black dotted boxes under PBCs:

$$\hat{H}^{nonrecip}_{SSH\,PBC'}(K) = \begin{bmatrix} 0 & t_1 & 0 & t_2e^{iK} & \kappa+i\gamma & 0 & 0 & 0 \\ t_1 & 0 & t_2 & 0 & 0 & \kappa-i\gamma & 0 & 0 \\ 0 & t_2 & 0 & t_1 & 0 & 0 & \kappa-i\gamma & 0 \\ t_2e^{-iK} & 0 & t_1 & 0 & 0 & 0 & 0 & \kappa+i\gamma \\ \kappa+i\gamma & 0 & 0 & 0 & 0 & t_1 & 0 & t_2e^{iK} \\ 0 & \kappa-i\gamma & 0 & 0 & t_1 & 0 & t_2 & 0 \\ 0 & 0 & \kappa-i\gamma & 0 & 0 & t_2 & 0 & t_1 \\ 0 & 0 & 0 & \kappa+i\gamma & t_2e^{-iK} & 0 & t_1 & 0 \end{bmatrix}, \text{(E4)}$$

$$\hat{H}^{GL}_{SSH\,PBC'}(K) = \begin{bmatrix} i\gamma & t_1 & 0 & t_2e^{iK} & \kappa & 0 & 0 & 0 \\ t_1 & -i\gamma & t_2 & 0 & 0 & \kappa & 0 & 0 \\ 0 & t_2 & -i\gamma & t_1 & 0 & 0 & \kappa & 0 \\ t_2e^{-iK} & 0 & t_1 & i\gamma & 0 & 0 & 0 & \kappa \\ \kappa & 0 & 0 & 0 & i\gamma & t_1 & 0 & t_2e^{iK} \\ 0 & \kappa & 0 & 0 & t_1 & -i\gamma & t_2 & 0 \\ 0 & 0 & \kappa & 0 & 0 & t_2 & -i\gamma & t_1 \\ 0 & 0 & 0 & \kappa & t_2e^{-iK} & 0 & t_1 & i\gamma \end{bmatrix}, \text{(E5)}$$

where K is the Bloch wave number, and t_1 and t_2 describe the in-layer nearest-neighbor couplings in the lattice. κ is the Hermitian isotropic interlayer hopping, and γ is the non-Hermitian part of the interlayer hopping in the passive waveguide arrays and gain or loss strength in the gain and lossy waveguides, respectively. Here, K, t_1, t_2 κ, γ are real numbers. The type2 similarity transformation between $\hat{H}^{nonrecip}_{SSH\,PBC'}(K)$ and $\hat{H}^{GL}_{SSH\,PBC'}(K)$ is $\hat{C}_{8'}\hat{H}^{GL}_{SSH\,PBC'}\hat{C}^{-1}_{8'} = \hat{H}^{nonrecip}_{SSH\,PBC'}$, and the transformation matrix is given by

$$\hat{C}_{8'} = \begin{bmatrix} a+ib & b\gamma & 0 & 0 & a-ib & -b\gamma & 0 & 0 \\ -b\gamma & a+ib & 0 & 0 & b\gamma & a-ib & 0 & 0 \\ 0 & 0 & a+ib & -b\gamma & 0 & 0 & a-ib & b\gamma \\ 0 & 0 & b\gamma & a+ib & 0 & 0 & -b\gamma & a-ib \\ a-ib & -b\gamma & 0 & 0 & a+ib & b\gamma & 0 & 0 \\ b\gamma & a-ib & 0 & 0 & -b\gamma & a+ib & 0 & 0 \\ 0 & 0 & a-ib & b\gamma & 0 & 0 & a+ib & -b\gamma \\ 0 & 0 & -b\gamma & a-ib & 0 & 0 & b\gamma & a+ib \end{bmatrix}, \text{(E6)}$$

where neither a nor b is 0. When $K = 0$, the eigenvalues of the eight periodic-boundary-condition energy bands are

$$E^{SSH'}_{\pm\pm\pm} = \pm t_2 \pm \kappa \pm \sqrt{{t_1}^2 - \gamma^2}, \text{(E7)}.$$

Fig. 12(c) compares the E–γ relation of $\hat{H}^{nonrecip}_{SSH\,PBC'}(K)$ (solid lines) and $\hat{H}^{GL}_{SSH\,PBC'}(K)$ (discrete circles) when $K = 0$, t_1= 2, t_2 = 1, and κ=0.2. Four EPs emerge at $\gamma = t_1$, marked by black dots. Two DPs of the fourth and fifth eigenvalues emerge at $\gamma = \sqrt{{t_1}^2 - (t_2+\kappa)^2}$ and $\gamma = \sqrt{{t_1}^2 - (t_2-\kappa)^2}$, marked by green dot and red dot, respectively. The DP of the third and the fourth eigenvalues and the DP of the fifth and the sixth eigenvalues emerge at $\gamma = \sqrt{{t_1}^2 - {t_2}^2}$, marked by orange dots.

In Figs. 12(d–i), we compare the bulk bands given by $\hat{H}^{nonrecip}_{SSH\ PBC'}(K)$ (solid lines) and $\hat{H}^{GL}_{SSH\ PBC'}(K)$ (discrete circles) with increasing γ. When γ = 0, the lattice is the Hermitian SSH model. When γ≠0, the edge DPs become EPs marked by pink and magenta crosses, and gradually move toward the center of FBZ, while the complex energy region expands from the edge to the center of the FBZ [Fig. 12(d)]. As γ increases, the real parts of bands gradually move toward zero energy, and the central gap is closed (marked by black crosses) when $\gamma = \sqrt{t_1{}^2 - (t_2+\kappa)^2}$ [Fig. 12(e)]. The bands approach and the complex energy region expands gradually to form two central DPs when $\gamma = \sqrt{t_1{}^2 - t_2{}^2}$ [Fig. 12(f)]. The real parts of bands form a central DP again when $\gamma = \sqrt{t_1{}^2 - (t_2-\kappa)^2}$ [Fig. 12(g)]. Two EPs merge into one EP at the center of the FBZ When $\gamma = t_1$ [Fig. 12(h)], and disappear when $\gamma > t_1$ [Fig. 12(i)].

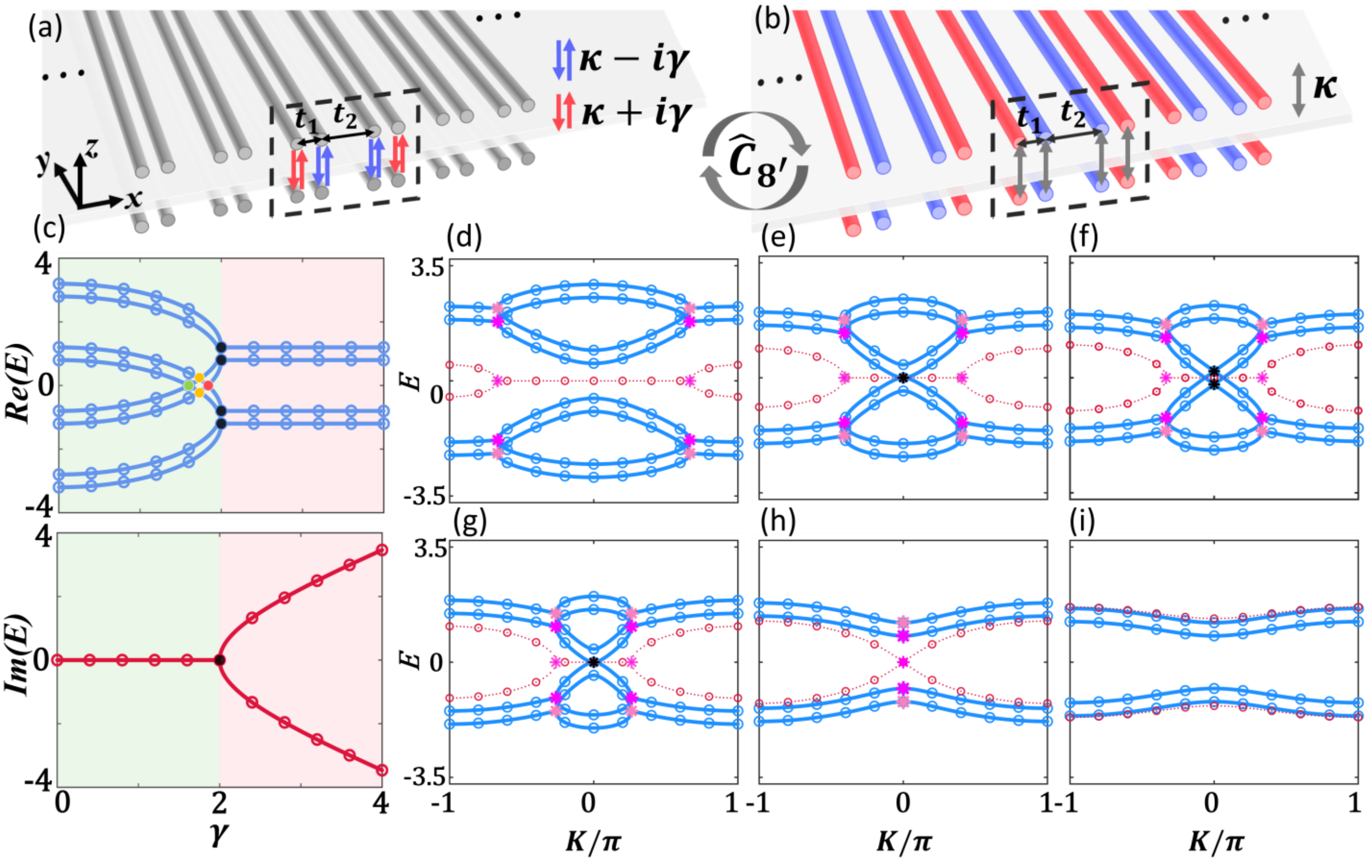

FIG. 12. Schematic of the BNH SSH model for (a) passive waveguide arrays with non-Hermitian interlayer coupling κ+iγ and κ-iγ and (b) gain (red) and lossy (blue) waveguides. (c) Comparison of real parts and imaginary parts of eigenvalues of $\hat{H}^{nonrecip}_{SSH\ PBC'}(K)$ (solid lines) and $\hat{H}^{GL}_{SSH\ PBC'}(K)$ (discrete circles) as a function of γ when $K = 0$. EPs are marked by black dots, and different DPs are marked by green, orange, and red dots, respectively. Comparison of the bulk band structures of BNH SSH model in the PBCs using

parameters t_1= 2, t_2= 1, κ=0.2 and γ = 1 for (d), γ = 1.6 for (e), γ =1.732 for (f), γ = 1.833 for (g), γ = 2 for (h), and γ = 2.4 for (i). The blue solid (red dot) lines indicate the real (imaginary) parts of eigenvalues of $\hat{H}^{nonrecip}_{SSH\,PBC'}(K)$, and the blue (red) discrete circles indicate the real (imaginary) parts of eigenvalues of $\hat{H}^{GL}_{SSH\,PBC'}(K)$.

With periodic (scattering) boundary conditions along the x (y) direction, the bilayer supercells of C_{6v} topologically nontrivial PC with zigzag-type parallel non-Hermitian domain walls constructed by NIC [Fig. 13(a)] and on-site gain/loss [Fig. 13(b)] are considered. Here we also take 40 unit cells along the y direction per layer. The Hamiltonians of the bilayer supercell using the tight-binding approximation in k space can be written as

$$\hat{H}^{nonrecip}_{C6\,PBC'}(K) = \hat{\sigma}_0 \otimes H_{mono} + \kappa \hat{\sigma}_x \otimes \hat{\sigma}_0 \otimes \hat{I}_{N/2} + i\gamma \hat{\sigma}_x \otimes \hat{\sigma}_z \otimes \hat{I}_{N/2}, \text{(E8)}$$

$$\hat{H}^{GL}_{C6\,PBC'}(K) = \begin{bmatrix} H_{GL} & \kappa \hat{I}_N \\ \kappa \hat{I}_N & H_{GL} \end{bmatrix}, \text{(E9)}$$

which satisfy $\hat{C}_{C6'} \hat{H}^{GL}_{C6\,PBC'} \hat{C}^{-1}_{C6'} = \hat{H}^{nonrecip}_{C6\,PBC'}$. The matrix forms of H_{mono} and H_{GL} are given in Eqs. (D1) and (D2). K is the Bloch wave number; $\hat{I}_N$ ($\hat{I}_{N/2}$) is $\mathrm{N} \times \mathrm{N}$ ($\frac{\mathrm{N}}{2} \times \frac{\mathrm{N}}{2}$) identical matrix, and here we take N=240. The non-Hermitian interlayer coupling is $\kappa + i\gamma$ ($\kappa - i\gamma$) between the first layer and the second layer in the upper (lower) half part of the y-axis as shown in Fig. 13(a). Each coupling term consists of a Hermitian part, κ, and a non-Hermitian part, γ. κ is the reciprocal interlayer nearest-neighbor hopping as shown in Fig. 13(b). Fig. 13(c) compares the E–γ relation of the eight DITISs of $\hat{H}^{nonrecip}_{C6\,PBC'}(K)$ (solid lines) and $\hat{H}^{GL}_{C6\,PBC'}(K)$ (discrete circles) with parameters $t_1 = 5$, $t_2 = 1$, $\kappa = 0.2$, and $K = 0$, which is similar to the E–γ relation of the BNH SSH model [Fig. 12(c)]. The non-Hermitian domain-induced bands are highlighted by dark and light blue (red) in real(imaginary) parts.

Figs. 13(d-h) compare the projected band structures given by $\hat{H}^{nonrecip}_{C6\,PBC'}(K)$ (solid lines) and $\hat{H}^{GL}_{C6\,PBC'}(K)$ (discrete circles) with increasing γ. The projected bands of the eight DITISs are similar to the bulk bands shown in Figs. 13(e-i). We conclude that the parallel non-Hermitian domain wall in C_{6v} PCs degenerates into the non-Hermitian SSH model shown in Fig. 12 if we just consider the sites and couplings near the bilayer domain walls. The non-Hermitian DITISs can be described by the non-Hermitian SSH model, whose topological index can also be defined in this way.

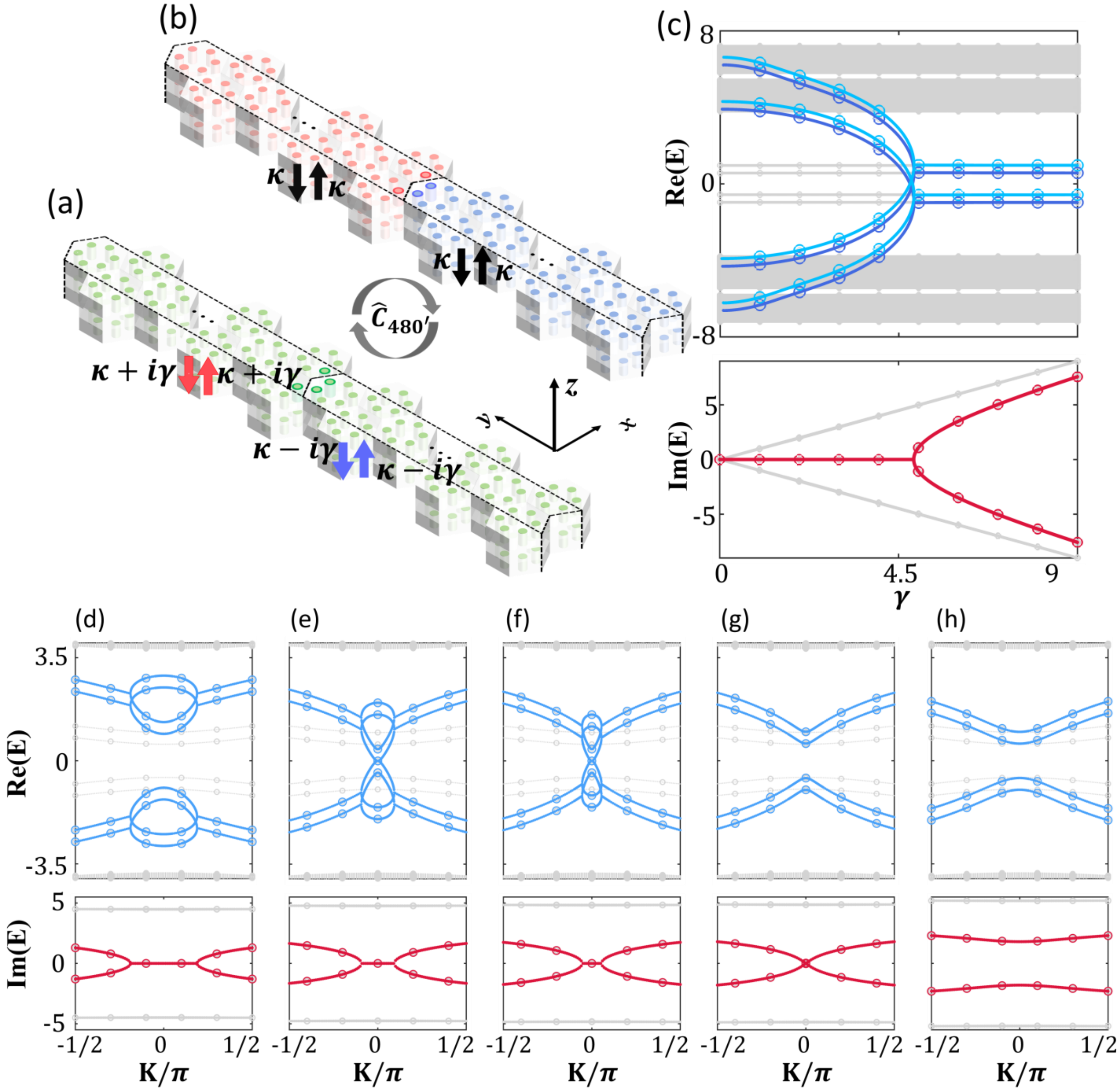

FIG. 13. (a) Supercell of bilayer C$_{6v}$ PCs with zigzag-type parallel non-Hermitian domain walls constructed by (a) anisotropic coupling strength $\kappa+i\gamma$ (red arrows) and $\kappa-i\gamma$ (blue arrows) between two layers, and (b) on-site gain (red) and loss (blue). (c) Comparison of the eigenvalues of $\hat{H}^{nonrecip}_{C6\,PBC'}(K)$ (solid line) and $\hat{H}^{GL}_{C6\,PBC'}(K)$ (discrete circles) with varying γ at $K=0$. The DITISs are colored in blue (real parts) and red (imaginary parts). Comparison of parts of the projected band structures of $\hat{H}^{nonrecip}_{C6\,PBC'}(K)$ (solid line) and $\hat{H}^{GL}_{C6\,PBC'}(K)$ (discrete circles) with $t_1=5$, $t_2=1$, κ=0.2 and γ = 4.5 for (d), γ = 4.782 for (e), γ =4.8468 for (f), γ = 4.8817 for (g), γ = 5.2 for (h).
